# Efficacy and Safety of Baxdrostat in Participants with CKD and Uncontrolled Hypertension: A Randomized, Double-Blind, Placebo-Controlled Trial

**DOI:** 10.1681/ASN.0000000849

**Published:** 2025-09-06

**Authors:** Jamie P. Dwyer, Noha Maklad, Ola Vedin, John Monyak, Robin Myte, Glenn M. Chertow, Hiddo J.L. Heerspink, Dustin J. Little

**Affiliations:** 1 Division of Nephrology and Hypertension, University of Utah, Salt Lake City, Utah; 2 Late Cardiovascular, Renal, and Metabolism, BioPharmaceuticals R&D, AstraZeneca, Gaithersburg, Maryland; 3 Late-Stage Development, Cardiovascular, Renal, and Metabolism, BioPharmaceuticals R&D, AstraZeneca, Gothenburg, Sweden; 4 Biometrics, Late-Stage Development, Cardiovascular, Renal and Metabolism, BioPharmaceuticals R&D, AstraZeneca, Gaithersburg, Maryland; 5 Departments of Medicine, Epidemiology and Population Health, and Health Policy, Stanford University School of Medicine, Stanford, California; 6 Department of Clinical Pharmacy and Pharmacology, University of Groningen, University Medical Center Groningen, Groningen, The Netherlands; 7 The George Institute for Global Health, Sydney, New South Wales, Australia

**Keywords:** aldosterone, CKD, chronic kidney disease, clinical trial, hypertension

## Abstract

**Key Points:**

- This phase 2 trial assessed baxdrostat, an aldosterone synthase inhibitor, in participants with CKD and uncontrolled hypertension.
- Baxdrostat showed placebo-corrected reduction in systolic BP of –8.1 (95% confidence interval, –13.4 to –2.8) mm Hg, *P* = 0.003.
- Baxdrostat was well tolerated; hyperkalemia was the most frequent treatment-emergent adverse event.

**Background:**

Aldosterone increases BP and contributes to CKD progression. We evaluated the efficacy and safety of baxdrostat, an aldosterone synthase inhibitor, in participants with CKD and uncontrolled hypertension.

**Methods:**

This was a phase 2, randomized, double-blind, placebo-controlled, multicenter trial (NCT05432167). Eligible participants were treated with an angiotensin-converting enzyme inhibitor or angiotensin receptor blocker and had a mean seated office systolic BP ≥140 mm Hg (without diabetes) or ≥130 mm Hg (with type 2 diabetes) and a urine albumin-creatinine ratio of≥100 mg/g. Participants were randomized (1:1:1) to baxdrostat low-dose (0.5 mg up-titrated to 1 mg), high-dose (2 mg up-titrated to 4 mg), or placebo for 26 weeks. The primary end point was change from baseline in mean seated office systolic BP at week 26 in the baxdrostat pooled treatment group versus placebo. The secondary end point assessed this change by high-dose or low-dose baxdrostat; end points were tested sequentially in a hierarchal manner.

**Results:**

Between April 29, 2022, and May 2, 2024, 195 participants were randomized. The mean (SD) age was 66 (11) years, 32% were women, 113 (58%) were White, and 80% had type 2 diabetes. The mean (SD) baseline systolic BP was 151.2 (13.1) mm Hg; the mean (SD) baseline eGFR was 44 (14) ml/min per 1.73 m^2^, and the median (Q1, Q3) urine albumin-creatinine ratio was 714 (307, 1429) mg/g. The mean placebo-corrected change in systolic BP from baseline to week 26 for the baxdrostat pooled group was –8.1 (95% confidence interval, −13.4 to −2.8; *P* = 0.003) mm Hg; low-dose −9.0 (−15.1 to −2.9; *P* = 0.004) mm Hg; high-dose −7.2 (−13.2 to −1.2; *P* = 0.02) mm Hg. Hyperkalemia was recorded as an adverse event in 41% (53/128) of participants in the baxdrostat pooled group and 5% (3/64) in the placebo group.

**Conclusions:**

Baxdrostat reduced systolic BP in participants with CKD and uncontrolled hypertension. Hyperkalemia was reported more commonly as an adverse event with baxdrostat versus placebo.

**Clinical Trial registry name and registration number:**

NCT05432167.

## Visual Abstract

**Figure:**
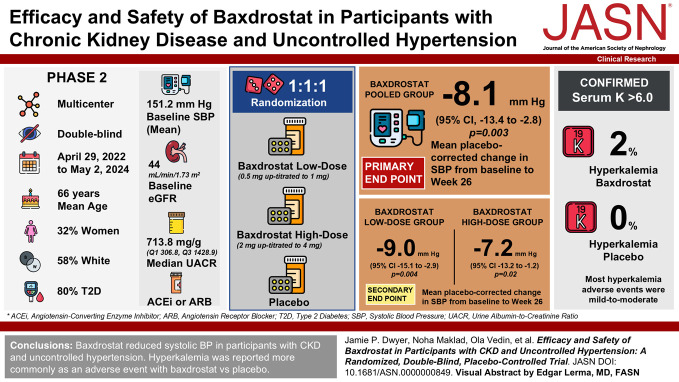

## Introduction

Hypertension persists as a global health burden^1–3^ and associates with a higher risk of cardiovascular events.^2,4–6^ Elevated systolic BP has been shown to be the leading modifiable risk factor for cardiovascular events and the leading risk factor for all deaths globally.^2,4,6–8^

Among patients with CKD, uncontrolled hypertension is one of the most common modifiable risk factors for cardiovascular events and CKD progression, regardless of the cause of CKD.^5,8^ It is estimated that >840 million people live with CKD worldwide, and up to 90% of this population also have hypertension.^5,9–11^ The majority of patients with CKD and hypertension do not achieve BP targets.^12–15^ In a recent World Health Organization report, 62% of deaths from CKD were attributed to elevated systolic BP.^6,9,11^

Aldosterone is implicated in the pathophysiology of hypertension and can lead to vascular calcification and fibrosis.^16–18^ Aldosterone stimulates renal sodium reabsorption in exchange for potassium in the distal nephron and can contribute to a decline in kidney function through renal inflammation and fibrosis.^5,19–23^ Mineralocorticoid receptor antagonists (MRAs) targeting this pathway do not fully inhibit the deleterious effects of aldosterone and may increase serum aldosterone concentrations.^18,24,25^ Highly selective aldosterone synthase inhibitors (ASIs) decrease aldosterone synthesis, providing an alternative strategy to inhibit aldosterone effects without affecting cortisol production.^26–28^

Baxdrostat is a potent and highly selective ASI previously shown to result in significant reductions in systolic BP without affecting cortisol in participants with treatment-resistant hypertension (BP ≥130/80 mm Hg and receiving at least three antihypertensive agents).^29^ This phase 2 study evaluated the efficacy and safety of baxdrostat compared with placebo in participants with CKD and uncontrolled hypertension.

## Methods

### Study Design

**Figure 1:**
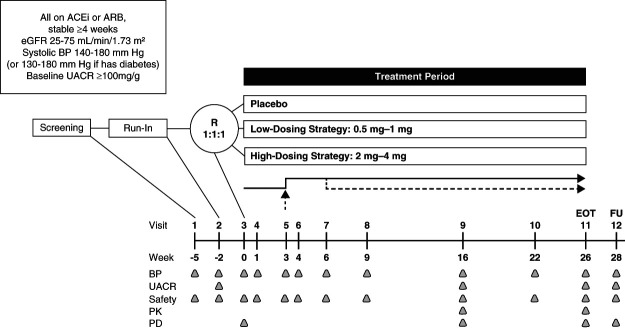
**Study design for the FigHTN trial.** ACEi, angiotensin-converting enzyme inhibitor; ARB, angiotensin receptor blocker; EOT, end of treatment; FU, follow-up; PD, pharmacodynamics; PK, pharmacokinetics; R, randomization; UACR, urinary albumin-to-creatinine ratio.

This was a phase 2, randomized, double-blind, placebo-controlled, multicenter, parallel-group, dose-ranging study conducted at 78 sites in the United States to evaluate the efficacy and safety of baxdrostat in adults with CKD and uncontrolled hypertension. We conducted this study in accordance with the principles of the Declaration of Helsinki and the International Council for Harmonisation Good Clinical Practice guidelines. Each site had approval from an institutional review board or ethics committee, and appropriate written informed consent was obtained from each participant before commencement of study procedures. The trial was registered with ClinicalTrials.gov (NCT05432167) and has been completed. Figure 1 shows the study design.

### Population

We enrolled participants aged ≥18 years with an eGFR (based on the 2009 CKD Epidemiology Collaboration equation) of 25–75 ml/min per 1.73 m^2^ (inclusive) at screening, a urine albumin-creatinine ratio (UACR) of ≥100 mg/g, and a mean seated office systolic BP between 140 and 180 mm Hg without diabetes or between 130 and 180 mm Hg with type 2 diabetes. Eligible participants were taking an angiotensin-converting enzyme (ACE) inhibitor or angiotensin receptor blocker (ARB) at the maximum tolerated daily dose for >4 weeks before screening.

Participants were excluded if they had type 1 diabetes, glycosylated hemoglobin >10.5% at screening, or were concomitantly treated with MRAs or potassium-sparing diuretics. Complete eligibility criteria are available in the Supplemental Material.

### Randomization and Masking

Following a 5-week screening period (including a mandatory 2-week run-in period), we randomly assigned participants in a 1:1:1 ratio into one of three treatment groups: baxdrostat low-dose, baxdrostat high-dose, or matching placebo for a 26-week treatment period, followed by a 2-week follow-up period. We stratified randomization by sodium-glucose cotransporter 2 (SGLT2) inhibitor use, baseline systolic BP ≤155 or>155 mm Hg, and eGFR ≤45 or >45 ml/min per 1.73 m^2^. Participants were randomized using a computer-generated, stratified, randomized block list (with three stratification factors and blocking within each stratum for a block size of six) implemented through an automated interactive response technology system. We capped the proportion of participants with an eGFR of 60–75 ml/min per 1.73 m^2^ at screening to ensure that this subpopulation did not exceed approximately 25% of the total population. Participants and all trial personnel (except the members of the independent data safety monitoring board) were blinded to randomized treatment assignments.

### Interventions and Outcomes

Oral baxdrostat 0.5, 1, 2, and 4 mg was administered once daily to participants randomized to active treatment. Participants randomized to the low-dose group initially received 0.5 mg and participants in the high-dose group initially received 2 mg. At week 3, the baxdrostat dose could be up-titrated to 1 mg for the low-dose group and to 4 mg for the high-dose group (see Supplemental Material). When approximately 95% of participants had been randomized, a protocol amendment took effect to define potassium and sodium up-titration thresholds (previously undefined). This allowed down-titration for previously up-titrated participants at any point during the study (previously only allowed from week 5 to week 8) and down-titration or dose interruption for potassium values of ≥6.0 mEq/L or confirmed ≥5.5 mEq/L within 72 hours (previously only for values ≥6.0). Doses of study drug could be up-titrated if participants had not achieved systolic BP <130 mm Hg and provided the participant had not experienced potassium ≥5.0 mEq/L, sodium <135.0 mEq/L, or an eGFR decrease of ≥30% from randomization. For participants who had been up-titrated at week 3, the dose could be down-titrated if the investigator considered electrolyte balance or declining kidney function to be a significant risk for the participant or if serum potassium was >5.0 mEq/L.

The primary end point was the change in mean seated office systolic BP from baseline to week 26 in participants receiving baxdrostat (pooled treatment) compared with placebo. The secondary end point was change from baseline to week 26 in mean seated office systolic BP in participants receiving high-dose or low-dose baxdrostat compared with placebo. The mean seated office systolic BP was defined as the average of three seated office systolic BP measurements at any single clinical site visit. Each site was provided with an automated oscillometric BP device (Automated Diagnostic Company E-Sphyg3). Seated office BP was obtained from the same arm using an appropriately sized cuff with the bladder centered over the brachial artery. After a 5-minute rest period, the mean BP was calculated and recorded from three measurements taken 1–2 minutes apart.

Exploratory end points for each dosing strategy included the proportion of participants achieving mean seated office systolic BP <130 mm Hg at week 26, change from baseline to week 26 in mean seated office diastolic BP, and change from baseline to week 26 in UACR. We also assessed change from baseline in eGFR to week 26 and the proportion of participants who experienced a >30% or >50% decline in eGFR from one visit to the next.

Safety and tolerability end points were treatment-emergent adverse events (TEAEs) and serious adverse events (TESAEs) from the time of randomization until the end of the follow-up period, regardless of treatment discontinuation. Adverse events of special interest (AESI) included hypotension and abnormalities in serum potassium and/or sodium laboratory concentrations that required clinical intervention. Additional safety end points included treatment-emergent laboratory abnormalities; change in serum potassium and sodium concentrations from baseline to week 26 for both baxdrostat low-dose and high-dose groups compared with placebo; confirmed elevated potassium values >5.5 and >6.0 mEq/L, defined as at least two consecutive test results above the threshold; vital signs; and clinical laboratory evaluations. A description of clinical and laboratory measurements is available in the Supplemental Material.

### Statistical Analyses

We calculated the sample size for the study, assuming an early withdrawal rate of 8%, to include approximately 174 participants, sufficient for 90% power for the primary end point assuming a treatment difference of 6 (SD 11) mm Hg between the pooled baxdrostat and placebo groups using a two-sided significance level of 0.05. All statistical analyses were performed using SAS version 9.4 or higher.

To protect the overall two-sided alpha level of 5%, the objectives for primary and secondary efficacy end points were tested sequentially in a hierarchical manner, in the following order: (*1*) change from baseline to week 26 in mean seated office systolic BP in pooled baxdrostat compared with placebo; (*2*) change from baseline to week 26 in mean seated office systolic BP in high-dose baxdrostat compared with placebo; and (*3*) change from baseline to week 26 in mean seated office systolic BP in low-dose baxdrostat compared with placebo. Only if a test was statistically significant (α 0.05) was the next test in the sequence performed.

For efficacy and safety end points, baseline was defined as the last nonmissing value obtained before the first dose/administration of baxdrostat, unless otherwise specified.

Efficacy analyses for the primary end point, change in systolic BP, were performed in the modified intent-to-treat (mITT) population, defined as all participants in the randomized treatment assignment (intent-to-treat population) who received at least one dose of any study drug. Intercurrent events of treatment discontinuation and initiation of other medications were handled using a treatment policy strategy, with all postevent data included and participants analyzed according to randomized assignment, while the intercurrent event of death was handled using a hypothetical strategy, with postdeath data treated as missing. We used a mixed model for repeated measures (MMRM) to perform these analyses, assuming an unstructured variance structure for the residual variance. The analysis included fixed effects for treatment, visit, stratification variables (SGLT2 inhibitor use and CKD category), and the treatment-by-visit interaction, along with a covariate of the corresponding baseline value and the baseline-by-visit interaction. We implicitly handled missing data in the parameter estimation, assuming values in participants with missing data were similar to those in participants with observed data having the same treatment assignment and covariate values (*i.e*., missing at random). We performed analyses for change in diastolic BP, UACR, and eGFR as above for change in systolic BP, except the stratification variable of baseline systolic BP was added as a covariate and UACR data were log-transformed. We derived percent change in UACR from the back-transformed geometric mean change from baseline to week 26. We analyzed achievement of systolic BP <130 mm Hg after 26 weeks of treatment using logistic regression with terms for treatment, baseline systolic BP value, and the abovementioned stratification variables. In this analysis, we assumed that participants with missing systolic BP values at week 26 had not achieved <130 mm Hg.

We summarized safety end points descriptively and included all participants who received at least one dose of study drug. We used MedDRA v27.0 to categorize adverse events (AE). TEAEs were defined as those AEs that newly occurred or worsened in severity during the double-blind treatment period. On-study AEs are reported, defined as any events that occurred after the first double-blind treatment.

## Results

### Procedural Characteristics and Adherence to Intervention

**Figure 2:**
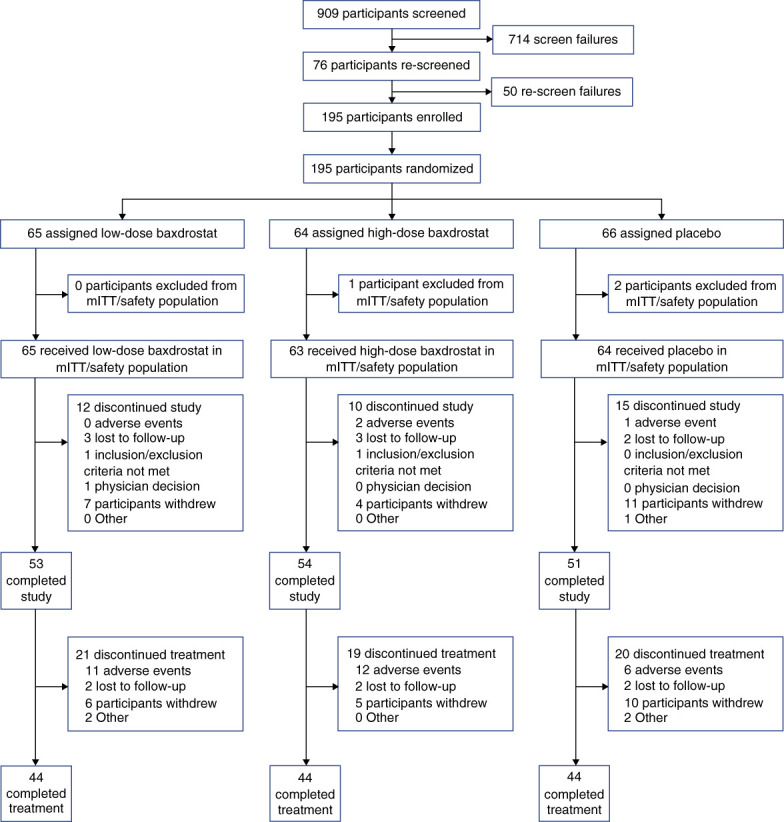
**Participant disposition.** Of 714 screen failures, 659 participants did not meet inclusion or exclusion criteria, five experienced an adverse event (before initiation of study drug), 11 were lost to follow-up, physician decision led to the withdrawal of two participants, 17 patients withdrew by own choice, one participant had COVID-19, and 19 participants were reported as “other.” Of 50 participants who failed rescreening, 44 failed to meet inclusion/exclusion criteria, one experienced an adverse event (before initiation of study drug), two participants withdrew by choice, one participant had COVID-19, and two participants were reported as “other.” Overall, 132 (68%) participants completed treatment. COVID-19, coronavirus disease 2019; mITT, modified intention to treat.

We conducted the study from April 29, 2022, to May 2, 2024. Of 909 participants screened, we enrolled 195 participants who were randomly assigned: three participants were excluded before treatment initiation and 192 participants comprised the mITT population and safety analysis sets (baxdrostat pooled treatment group, *n*=128 [high-dose, *n*=63; low-dose, *n*=65], placebo, *n*=64; Figure 2). Overall, 132 (68%) participants completed treatment; the main reasons for treatment discontinuation were “adverse event” and “withdrawal by participant.”

### Participants

Baseline demographics and clinical characteristics were similar between groups (intent-to-treat population, *N*=195; Table 1); overall, 63 (32%) were women, 113 (58%) were White, and 156 (80%) had type 2 diabetes. The mean (SD) age was 66 (11) years, and the mean (SD) systolic BP and diastolic BP were 151.2 (13.1) mm Hg and 81.1 (10.2) mm Hg, respectively, despite 167 (86%) participants taking two or more antihypertensive medications. The mean (SD) baseline eGFR was 44 (14) ml/min per 1.73 m^2^; the median (Q1, Q3) UACR was 714 (307, 1429) mg/g, and 149 (76%) participants had UACR >300 mg/g.

**Table 1:** Participant demographics and characteristics by treatment group at baseline (intention-to-treat population)

Participant Demographic or Characteristic	Baxdrostat Low-Dose *n*=65	Baxdrostat High-Dose *n*=64	Baxdrostat Treatment Groups Pooled *n*=129	Placebo *n*=66	Total *N*=195
Age, yr, mean (SD)	67 (13)	67 (11)	67 (12)	66 (11)	66 (11)
**Sex, *n* (%)**					
Male	42 (65)	43 (67)	85 (66)	47 (71)	132 (68)
Female	23 (35)	21 (33)	44 (34)	19 (29)	63 (32)
**Ethnic origin, *n* (%)**					
Hispanic or Latino	24 (37)	16 (25)	40 (31)	26 (39)	66 (34)
**Race, *n* (%)**					
American Indian or Alaska Native	0	0	0	1 (2)	1 (1)
Asian	4 (6)	3 (5)	7 (5)	5 (8)	12 (6)
Black or African American	19 (29)	24 (38)	43 (33)	20 (30)	63 (32)
Multiple	0	1 (2)	1 (0.8)	0	1 (1)
Native Hawaiian or other Pacific Islander	0	0	0	1 (2)	1 (1)
Not reported	1 (2)	2 (3)	3 (2)	1 (2)	4 (2)
White	41 (63)	34 (53)	75 (58)	38 (58)	113 (58)
BMI, kg/m^2^, mean (SD)	31.1 (6.3)	31.0 (6.3)	31.0 (6.3)	31.7 (6.4)	31.3 (6.3)
Systolic BP, mm Hg, mean (SD)	150.7 (13.3)	151.0 (13.0)	150.9 (13.1)	151.9 (13.1)	151.2 (13.1)
Diastolic BP, mm Hg, mean (SD)	80.8 (9.5)	80.8 (9.9)	80.8 (9.7)	81.7 (11.1)	81.1 (10.2)
**eGFR, ml/min per 1.73 m** ^**2**^ **, mean (SD)**	46 (15)	44 (14)	45 (14)	44 (15)	44 (14)
<30 ml/min per 1.73 m^2^, *n* (%)	8 (12)	9 (14)	17 (13)	17 (26)	34 (17)
30–<45 ml/min per 1.73 m^2^, *n* (%)	28 (43)	24 (37)	52 (40)	18 (27)	70 (36)
45–<60 ml/min per 1.73 m^2^, *n* (%)	15 (23)	23 (36)	38 (29)	18 (27)	56 (29)
≥60 ml/min per 1.73 m^2^, *n* (%)	14 (21)	8 (12)	22 (17)	13 (20)	35 (18)
**Potassium, mmol/L, mean (SD)**	4.4 (0.4)	4.4 (0.5)	4.4 (0.5)	4.3 (0.4)	4.4 (0.4)
≤4.0 mmol/L, *n* (%)	11 (17)	9 (14)	20 (16)	17 (26)	37 (19)
>4.0–4.5 mmol/L, *n* (%)	30 (46)	32 (50)	62 (48)	29 (44)	91 (47)
>4.5–4.8 mmol/L, *n* (%)	15 (23)	14 (22)	29 (22)	12 (18)	41 (21)
>4.8 mmol/L, *n* (%)	9 (14)	9 (14)	18 (14)	8 (12)	26 (13)
**Serum aldosterone, ng/dl,**^a^ **mean (SD)**	6.0 (5.2)	6.8 (5.9)	6.4 (5.5)	6.7 (6.3)	6.5 (5.8)
<6 ng/dl, *n* (%)	40 (62)	34 (53)	74 (57)	41 (62)	115 (59)
≥6 ng/dl, *n* (%)	23 (35)	29 (45)	52 (40)	22 (33)	74 (38)
**PRA,**^b^ **μg/L per h, mean (SD)**	2.7 (2.9)	3.1 (4.6)	2.9 (3.8)	3.1 (3.8)	2.9 (3.8)
<1 μg/L per h, *n* (%)	22 (34)	23 (36)	45 (35)	21 (32)	66 (34)
≥1 μg/L per h, *n* (%)	39 (60)	37 (58)	76 (59)	40 (61)	116 (59)
ARR,^b^ ng/dl per ng/ml per hr, mean (SD)	8.5 (18.7)	7.0 (9.3)	7.8 (14.8)	10.7 (20.5)	8.8 (16.9)
**UACR,**^c^ **mg/g, mean (SD)**	1291 (1624)	1035 (1157)	1164 (1412)	1188 (1093)	1172 (1310)
Microalbuminuria, *n* (%)	18 (28)	16 (25)	34 (26)	12 (18)	46 (24)
Macroalbuminuria, *n* (%)	47 (72)	48 (75)	95 (74)	54 (82)	149 (76)
Type 2 diabetes, *n* (%)	52 (80)	45 (70)	97 (75)	59 (89)	156 (80)
Heart failure, *n* (%)	1 (2)	2 (3)	3 (2)	3 (5)	6 (3)
**Use of ACEi/ARB, *n* (%)**					
ACEi only	23 (35)	25 (39)	48 (37)	21 (32)	69 (35)
ARB only	40 (62)	38 (59)	78 (60)	40 (61)	118 (61)
Both	0	0	0	1 (2)	1 (1)
SGLT2 inhibitor use, *n* (%)	21 (32)	21 (33)	42 (33)	22 (33)	64 (33)
Diuretic use, *n* (%)	30 (46)	24 (38)	54 (42)	31 (47)	85 (44)
GLP-1 RA use, *n* (%)	12 (19)	5 (8)	17 (13)	17 (26)	34 (17)
**Number of background antihypertensive medication use at baseline,**^d^ ***n* (%)**					
0	0	1 (2)	1 (0.8)	2 (3)	3 (2)
1	12 (18)	4 (6)	16 (12)	9 (14)	25 (13)
2	11 (17)	20 (31)	31 (24)	20 (30)	51 (26)
3	23 (35)	20 (31)	43 (33)	19 (29)	62 (32)
>3	19 (29)	19 (30)	38 (29)	16 (24)	54 (28)

Intention-to-treat population included all participants randomly assigned to the study. ACEi, angiotensin-converting enzyme inhibitors; ARB, angiotensin receptor blockers; ARR, aldosterone:renin ratio; BMI, body mass index; GLP-1 RA, glucagon-like peptide-1 receptor agonists; PRA, plasma renin activity; SGLT2, sodium-glucose cotransporter 2; UACR, urine albumin-creatinine ratio.

a It is assumed all sample collections were in sitting position: adult reference ranges: upright 8:00–10:00 am: ≤28 ng/dl, upright 4:00–6:00 pm: ≤21 ng/dl; number of participants with a serum aldosterone measurement were *n*=63 each in low-dose, high-dose, and placebo groups.

b Number of participants with a plasma renin activity or aldosterone-renin ratio measurement were *n*=61 in the low-dose group, *n*=60 in the high-dose group, and *n*=61 in the placebo group.

c Urine albumin-creatinine ratio was defined as normal: <30 mg/g; microalbuminuria: 30–300 mg/g; macroalbuminuria: >300 mg/g.

d Number of background antihypertensive medications other than angiotensin-converting enzyme inhibitors and angiotensin receptor blockers.

### Primary Outcome

**Figure 3:**
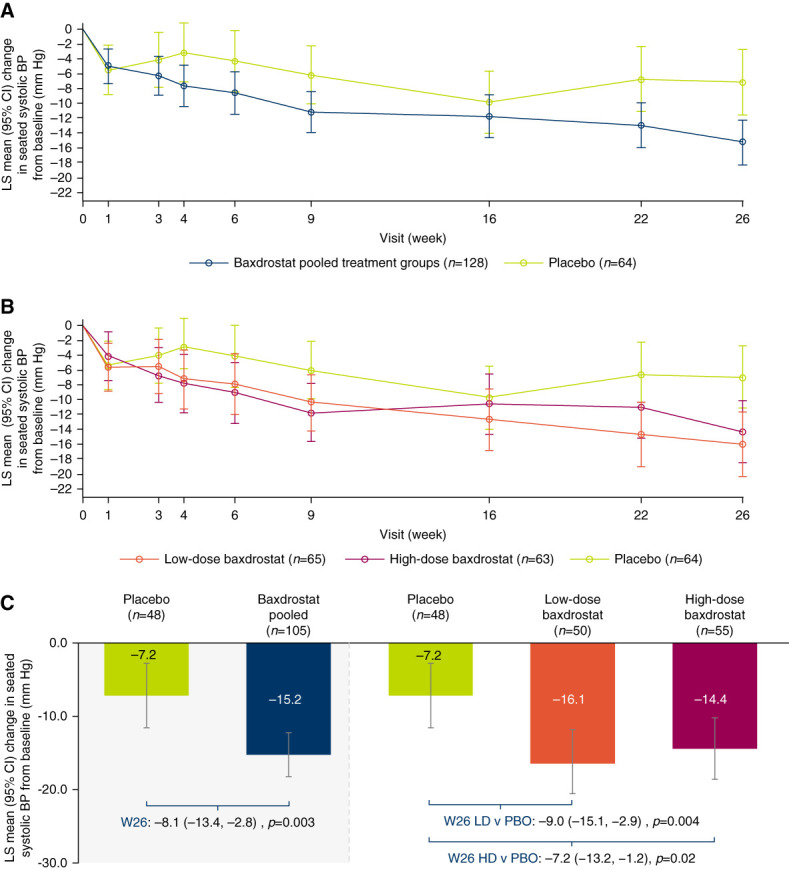
**Baxdrostat reduces seated systolic BP versus placebo.** LS mean (95% CI) change from baseline to week 26 in seated office systolic BP over time (line plots) in the (A) pooled baxdrostat treatment group (primary outcome), (B) baxdrostat low-dose and high-dose groups (secondary outcome), and (C) compared with placebo (bar graphs; primary and secondary outcomes). (C) Data below columns are percent change in LS mean versus placebo (95% CI). LS means for baxdrostat pooled group and placebo are from a MMRM including fixed effects for treatments (baxdrostat treatment groups versus placebo), visit, stratification variables (SGLT2 inhibitor use and CKD category), and the treatment-by-visit interaction, along with a covariate of the baseline seated office systolic BP value and the baseline seated office systolic BP-by-visit interaction. CI, confidence interval; HD, high-dose; LD, low-dose; LS, least squares; MMRM, mixed-effect model for repeated measures; PBO, placebo; SGLT2, sodium-glucose cotransporter 2; W, week.

Treatment with baxdrostat lowered seated office systolic BP, with reductions compared with placebo noted as early as week 3 and persisted through week 26 (Figure 3A). The mean (SEM) change in systolic BP from baseline to week 26 was –15.2 (1.5) mm Hg for the pooled baxdrostat group compared with –7.2 (2.2) mm Hg for placebo. Baxdrostat (pooled treatment group) resulted in a least squares (LS) mean change in seated office systolic BP from baseline to week 26 of –8.1 (95% confidence interval [CI], −13.4 to −2.8) mm Hg, *P* = 0.003 (Figure 3C) versus placebo.

These results were consistent across subgroups by baseline SGLT2 inhibitor treatment and eGFR (Supplemental Table 1).

### Secondary Outcomes

Treatment with baxdrostat low-dose and high-dose resulted in LS mean changes in systolic BP from baseline to week 26 of −9.0 (−15.1 to −2.9) mm Hg, *P* = 0.004, and −7.2 (−13.2 to −1.2) mm Hg, *P* = 0.02 (Figure 3, B and C), respectively, versus placebo.

### Exploratory Outcomes

Exploratory outcomes are tabulated in Supplemental Table 2. Systolic BP <130 mm Hg at week 26 was achieved by 38/105 (36%) participants in the pooled baxdrostat group, 21/50 (42%) in the low-dose group, and 17/55 (31%) in the high-dose group, compared with 8/48 (17%) of participants in the placebo group. The mean absolute change in seated office diastolic BP from baseline to week 26 was –5.6 (SD 10.2) mm Hg for the baxdrostat pooled group (low-dose group, –6.5 [11.4] mm Hg; high-dose group, –4.9 [9.0] mm Hg) and –1.0 (8.4) mm Hg for placebo. LS mean difference in diastolic BP for the pooled, low-dose, and high-dose groups was −4.8 (95% CI, −7.9 to −1.8) mm Hg, −5.0 (−8.6 to −1.5) mm Hg, and −4.7 (−8.2 to −⁠1.1) mm Hg, respectively, versus placebo.

**Figure 4:**
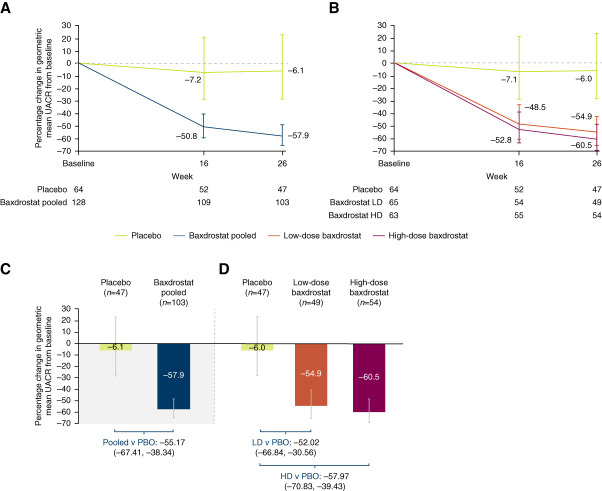
**Baxdrostat may reduce albuminuria versus placebo.** Percentage change from baseline to week 26 in geometric mean UACR over time (line graphs; exploratory outcome) and compared with placebo (bar graphs) in the (A and C) baxdrostat pooled group and (B and D) baxdrostat low-dose and high-dose groups. Line graphs: Baseline was defined as the geometric mean of the triplicate samples returned on week 2. Bar graph: Data below columns are percent change in geometric mean versus placebo (95% CI). Analyses were performed using a MMRM including fixed effects for treatment (baxdrostat pooled treatment groups or high-dose and low-dose groups versus placebo), stratification variables (SGLT2 inhibitor use, systolic BP category, and CKD category), visit, and the treatment-by-visit interaction, along with a covariate of the log-transformed baseline UACR value and the log-transformed baseline UACR-by-visit interaction. Log-transformed UACR values were used in the MMRM model; results were back-transformed to calculate geometric mean ratio, and percentage change from baseline was calculated as (GMR−1)×100. GMR, geometric mean ratio.

Percentage change in UACR from baseline to week 26 was −55.2% (95% CI, −67.4 to −38.3) for participants in the baxdrostat pooled group and −52.0% (−66.8 to −30.6) and −58.0% (−70.8 to −39.4) in the low-dose and high-dose groups, respectively, versus placebo (Figure 4).

Change in eGFR from baseline to week 26 was −2.3 (95% CI, −5.1 to 0.5) ml/min per 1.73 m^2^ for participants in the baxdrostat pooled group and −1.8 (−5.0 to 1.4) ml/min per 1.73 m^2^ and −2.8 (−6.0 to 0.4) ml/min per 1.73 m^2^ in the low-dose and high-dose groups, respectively, versus placebo (Supplemental Figure 1, A and B). Nineteen (15%) participants treated with baxdrostat had a >30% reduction in eGFR from one visit to the next (low-dose group ten [15%], high-dose group nine [14%]), compared with 11 (17%) placebo-treated participants. Reduction in eGFR of >50% from one visit to the next was noted in one participant randomized to placebo and zero participants randomized to baxdrostat.

### Pharmacodynamic Effects

Serum aldosterone concentrations were reduced, and serum renin levels were increased with baxdrostat treatment (Supplemental Table 3).

### Safety

TEAEs were experienced by 100 (78%) participants in the baxdrostat pooled group (low-dose group, 48 [74%]; high-dose group, 52 [83%]) and by 35 (55%) participants in the placebo group (Table 2). At least one TESAE was reported by 12 of 128 (9%) participants in the baxdrostat pooled group (low-dose group, seven [11%]; high-dose group, five [8%]) and two (3%) in the placebo group. Twenty-one (16%) baxdrostat participants experienced a TEAE leading to discontinuation of treatment (low-dose group, ten [15%]; high-dose group, 11 [17%]), compared with five (8%) placebo participants. There were no deaths reported during the study.

**Table 2:** Summary of adverse events on-study (safety outcomes)

TEAE/TESAE Category,^a^ *n* (%); (No. of Occurrences)^b^	Baxdrostat Low-Dose *n*=65	Baxdrostat High-Dose *n*=63	Baxdrostat Treatment Groups Pooled *n*=128	Placebo *n*=64	Total *N*=192
**Any TEAE**	48 (74; 165)	52 (83; 171)	100 (78; 336)	35 (55; 119)	135 (70; 455)
Treatment-related^c^	23 (35; 55)	31 (49; 69)	54 (42; 124)	14 (22; 20)	68 (35; 144)
**Any TESAEs**	7 (11; 14)	5 (8; 11)	12 (9; 25)	2 (3; 3)	14 (7; 28)
Treatment-related^c^	2 (3; 4)	1 (2; 1)	3 (2; 5)	1 (2; 2)	4 (2; 7)
Any TEAE or TESAE leading to death	0	0	0	0	0
Any TEAE leading to treatment discontinuation	10 (15; 13)	11 (17; 20)	21 (16; 33)	5 (8; 8)	26 (14; 41)
Any TESAE leading to treatment discontinuation	2 (3; 5)	2 (3; 3)	4 (3; 8)	1 (2; 1)	5 (3; 9)
Treatment-emergent AESIs^d^	11 (17; 16)	12 (19; 16)	23 (18; 32)	4 (6; 4)	27 (14; 36)
**Most common TEAEs (reported in ≥15% of patients)**					
Hyperkalemia	21 (32; 32)	32 (51; 53)	53 (41; 85)	3 (5; 4)	56 (29; 89)
Blood creatinine increased	11 (17; 14)	15 (24; 20)	26 (20; 34)	5 (8; 7)	31 (16; 41)

AE, adverse event; AESI, adverse event of special interest; TEAE, treatment-emergent adverse event; TESAE, treatment-emergent serious adverse event.

a Participants with multiple events in the same category are counted only once in that category. Participants with events in more than one category are counted once in each of those categories.

b The number of individual occurrences of the event.

c Includes both related and possibly related adverse events, as assessed by the investigator. Treatment-emergent adverse event on-study: all adverse events that newly occur or worsen in severity on or after the first double-blind treatment period on visit 3 (day 1). Death includes any death after the first double-blinded treatment during the study.

d Adverse events of special interest were hyperkalemia and hypotension.

Hyperkalemia was the most common TEAE, occurring in 53 (41%) participants in the baxdrostat pooled group (low-dose group, 21 [32%]; high-dose group, 32 [51%]) and three (5%) in the placebo group. Of the 53 participants in the baxdrostat pooled group who experienced a hyperkalemia AE, 27 events were reported by the investigator as mild (low-dose group, 13; high-dose group, 14); 21 were reported as moderate (low-dose group, six; high-dose group, 15), and five were reported as severe (low-dose group, two; high-dose group, three). One of the three hyperkalemia AEs in the placebo group was reported as mild in severity, and the other two as moderate. Three participants experienced a serious adverse event of hyperkalemia (two [3%] participants in the low-dose group and one [2%] in the high-dose group). Nine (7%) participants discontinued treatment due to hyperkalemia in the baxdrostat pooled group (four [6%] low-dose group, five [8%] high-dose group) versus one (2%) in the placebo group. The second most common TEAE was “blood creatinine increased,” reported in 26 (20%) participants in the baxdrostat pooled group (low-dose group, 11 [17%]; high-dose group, 15 [24%]) and five (8%) participants in the placebo group. None of the events described as “blood creatinine increased” required hospitalization or were otherwise considered serious adverse events. There were no AEs of adrenal insufficiency reported during the trial.

The AESI hyperkalemia was reported in 22 (17%) participants in the baxdrostat pooled group (low-dose group, ten [15%]; high-dose group, 12 [19%]) and two (3%) participants in the placebo group. Hypotension as an AESI occurred in two (2%) participants in the baxdrostat pooled group (two [3%] in the low-dose group and zero in the high-dose group) and one (2%) participant in the placebo group. No AESIs of hyponatremia were reported during the study.

**Figure 5:**
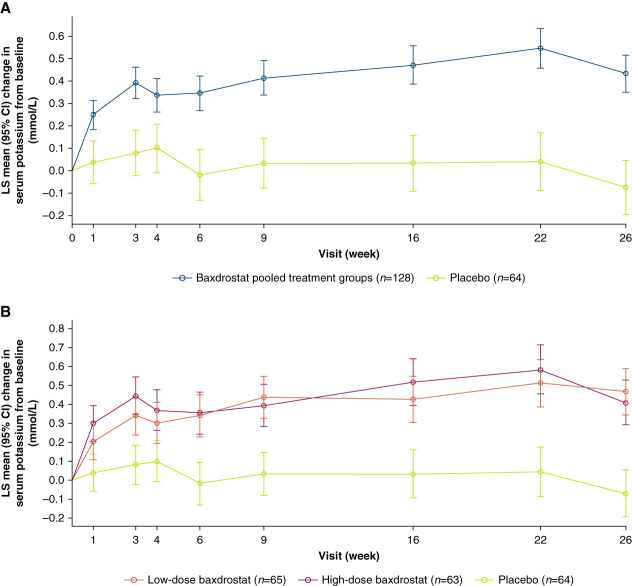
**Changes in potassium, with baxdrostat versus placebo.** LS means (95% CI) change from baseline to week 26 in serum potassium for (A) baxdrostat pooled group versus placebo and (B) baxdrostat low-dose and high-dose groups versus placebo (safety outcome). LS means are from a MMRM including fixed effects for treatment (baxdrostat treatment groups versus placebo), visit, stratification variables (SGLT2 inhibitor use, baseline systolic BP category, and CKD category), the treatment-by-visit interaction, along with a covariate of baseline potassium value and the baseline potassium-by-visit interaction.

An increase in mean serum potassium from baseline to week 26 was observed (Figure 5). Forty-seven (37%) and 14 (11%) baxdrostat pooled group participants had at least one serum potassium value of >5.5 mEq/L and >6.0 mEq/L, respectively (low-dose group, 23 [35%] and nine [14%]; high-dose group, 24 [38%] and five [8%]), compared with three (5%) and zero participants in the placebo group, respectively. Confirmed serum potassium concentrations >5.5 and >6.0 mEq/L were noted in 20 (16%) and two (2%) pooled baxdrostat participants, respectively, compared with zero placebo participants. The results for treatment-emergent abnormalities using potassium thresholds of ≥5.5 and ≥6.0 mEq/L are presented in Supplemental Table 4.

## Discussion

We designed the phase 2 FigHTN study to assess the efficacy and safety of baxdrostat in participants with CKD and uncontrolled hypertension. Baxdrostat demonstrated statistically significant reductions in systolic BP at week 26 compared with placebo, with low-dosing and high-dosing strategies, accompanied by reductions in diastolic BP and UACR. Baxdrostat was well tolerated with no unexpected safety findings during the study. Rates of hyperkalemia were higher in participants treated with baxdrostat, as expected due to the mechanism of action of the drug. The investigators considered most reported AEs to be nonserious.

Aldosterone contributes to CKD progression *via* the promotion of inflammation and fibrosis, and higher serum aldosterone concentrations have been independently associated with kidney failure.^22,23,30,31^ Challenges around the development of ASIs have centered around the similarity between aldosterone synthase (encoded by CYP11B2) and 11-*β*-hydroxylase (encoded by CYP11B1), which is involved in cortisol synthesis.^32^ Baxdrostat is a potent inhibitor of aldosterone synthase, previously shown not to meaningfully impact cortisol synthesis^33^; preclinical and phase 1 studies demonstrated that baxdrostat has higher selectivity for aldosterone synthase (CYP11B2) compared with 11*β*-hydroxylase (selectivity ratio, 100:1).^27,29,32,33^ Accordingly, no adrenal insufficiency events were noted in this study.

Despite similar rates of treatment completion in each treatment group, this study did not demonstrate clear differences in placebo-corrected changes in systolic BP or UACR between baxdrostat dosing groups. Numerically greater reductions in systolic BP were noted in the low-dose baxdrostat group and numerically greater UACR reductions in the high-dose group. These could be chance findings, as this study was not powered to assess differences between the 1 mg and 2 mg doses. It is also possible that the maximum dose response for systolic BP is achieved with 1 mg and that the UACR dose response curve is shifted to the right with higher doses showing larger effects. BP responses between the low-dose and high-dose baxdrostat treatment groups were different to those observed in a phase 2 trial of baxdrostat in participants with treatment resistant hypertension (BrigHTN),^29^ where treatment with baxdrostat for 12 weeks resulted in larger placebo-corrected changes with the 2 mg dose (–11.0 mm Hg; *P* < 0.001) compared with the 1 mg dose (−8.1 mm Hg; *P* = 0.003), with no statistically significant reduction in systolic BP observed with the 0.5 mg dose. A key difference between the two trials is that all participants in FigHTN had CKD, whereas CKD (eGFR <60 ml/min per 1.73 m^2^) was evident in 39/275 (14%) of BrigHTN participants. However, in a phase 1 study, systemic exposure or clearance of baxdrostat was not dependent on kidney function.^34^ In the context of the FigHTN results and the higher risk for hyperkalemia in patients with CKD, two ongoing phase 3 studies of baxdrostat in combination with dapagliflozin in participants with CKD and hypertension (NCT06268873 and NCT06742723)^35,36^ are using a starting baxdrostat dose of 1 mg in all participants, with potential up-titration to 2 mg according to serum potassium concentrations.

Despite differences in study populations and trial designs, findings from FigHTN are consistent with published studies of other ASIs. Vicadrostat has been evaluated for the treatment of patients with CKD in a phase 2 trial.^26^ The percentage change in UACR from baseline to week 14 (primary end point) was −39% with vicadrostat 10 mg monotherapy and −46% with vicadrostat when given on top of empaglifozin (post–second randomization), versus −3% and −11% with placebo, respectively.^26^ Finerenone, a nonsteroidal MRA, has shown placebo-corrected UACR reductions up to 38%^18,37^ and additive reductions in UACR when combined with the SGLT2 inhibitor empagliflozin.^38^ Eplerenone has shown mean UACR reductions from baseline up to 33%.^39^

In this study, placebo-corrected reductions in UACR of 55% were observed with baxdrostat monotherapy. According to a large meta-analysis of clinical trials including 29,979 participants followed for a median of 3.4 years, UACR reductions to the magnitude seen in FigHTN predict clinically relevant reductions in risk of clinical kidney events such as kidney failure.^40^ While BP lowering can reduce UACR, the magnitude of UACR reductions seen with baxdrostat suggests that baxdrostat may lower UACR through BP-dependent and BP-independent pathways. In this trial, change from baseline to week 26 in systolic BP was −7.2 mm Hg for placebo compared with −15.2 mm Hg for pooled baxdrostat, with corresponding UACR changes from baseline of −6.1% and −57.9%, respectively. Accordingly, in the subgroup of trials testing intensive versus standard BP control in the UACR meta-analysis, the intensive BP group achieved a 16% reduction in UACR compared with the standard BP group, a substantially smaller effect than observed here.^40^

Increases in serum potassium are consistent with the mechanism of action of drugs that inhibit aldosterone activity, including MRAs and ASIs. Generally, greater efficacy with these agents stemming from larger effects on aldosterone activity would be expected to associate with larger effects on serum potassium. In *post hoc* analyses of MRA trials in participants with heart failure, clinical benefits including reductions in mortality and heart failure events with MRA versus placebo were maintained among individuals with high on-treatment serum potassium concentrations.^41,42^ In this study, elevated serum potassium concentrations were typically transient, with the majority not confirmed on repeat testing. The addition of an SGLT2 inhibitor to an ASI may lower the risk of hyperkalemia^43–45^ and is an avenue for future research.

Initial reductions in eGFR were observed with baxdrostat treatment, as expected. After this initial decline, eGFR values stabilized, and the difference in eGFR at 26 weeks compared with placebo was modest. Numerically more “blood creatinine increases” were reported for participants randomized to baxdrostat, but all such AEs were deemed nonserious, and rates of 30% reduction in eGFR from one visit to the next (prespecified to evaluate for potentially clinically relevant acute or sub-acute reductions in eGFR) were numerically lower with baxdrostat compared with placebo.

The study is limited by a relatively small number of participants. Ambulatory BP monitoring was not assessed in this study; ambulatory BP monitoring is the primary end point in an ongoing placebo-controlled baxdrostat trial in participants with treatment-resistant hypertension (NCT06168409).^46^ In addition, changes to baxdrostat titration rules were made relatively late in the study, limiting our ability to assess the effect of baxdrostat on potassium when titrated using a more defined and conservative algorithm.

Baxdrostat is currently being studied in combination with dapagliflozin in two phase 3 double-blind randomized controlled trials for participants with CKD and hypertension. NCT06268873^35^ is evaluating baxdrostat/dapagliflozin in CKD progression (eGFR slope), while NCT06742723^36^ is assessing the effect of baxdrostat/dapagliflozin on cardiovascular and kidney events. In addition, NCT06677060^47^ will evaluate baxdrostat/dapagliflozin in lowering the risk of cardiovascular mortality and incident heart failure (with and without CKD).

In conclusion, in this phase 2 trial, baxdrostat at low (0.5–1 mg) and high (2–4 mg) doses was shown to be superior to placebo in reducing seated office systolic BP after 26 weeks of treatment in participants with CKD and uncontrolled hypertension, with UACR reductions of more than 50% from baseline compared with placebo. The safety profile of baxdrostat was consistent with the mechanism of action of the drug, and there were no new safety findings in this study.

## Supplementary Material

## Data Availability

Data belong to a third party, and authors are not authorized to share the data. Identity of Third Party: AstraZeneca. Data underlying the findings described in this manuscript can be requested in accordance with AstraZeneca's data sharing policy described online at https://astrazenecagrouptrials.pharmacm.com/ST/Submission/Disclosure. Data for studies directly listed on Vivli can be requested through Vivli at https://www.vivli.org. Data for studies not listed on Vivli can be requested through Vivli at https://vivli.org/members/enquiries-about-studies-not-listed-on-the-vivli-platform/. AstraZeneca Vivli member page is also available outlining further details: https://vivli.org/ourmember/astrazeneca.

## Declarative Statements

This study includes clinical experimentation and received Institutional Review Board or Ethics Committee approval. All patients provided written informed consent. This study includes clinical experimentation and complies with the Declaration of Helsinki.

## Supplemental Material

Supplemental Figure 1. LS means (95% CI) change from baseline to week 26 in eGFR over time (exploratory outcome) in the (A) pooled baxdrostat treatment group and (B) low-dose and high-dose baxdrostat groups compared with placebo.

Supplemental Table 1. LS mean change from baseline to week 26 in seated systolic BP for participants receiving baxdrostat (pooled) versus placebo stratified by SGLT2 inhibitor use and eGFR (mITT population; exploratory subanalysis).

Supplemental Table 2. Exploratory outcomes.

Supplemental Table 3. Percentage change from baseline to week 26 in geometric mean serum aldosterone and serum renin over time in the low- and high-dose baxdrostat groups compared with placebo (exploratory pharmacodynamic subanalysis).

Supplemental Table 4. Incidence of treatment-emergent serum potassium abnormalities (safety outcome).

Statistical Analysis Plan
