# Supplementary material for: Efficacy and Safety of Baxdrostat in Participants with CKD and Uncontrolled Hypertension: A Randomized, Double-Blind, Placebo-Controlled Trial
Source: J Am Soc Nephrol. 2025 Sep 6;37(2):299–311. doi: 10.1681/ASN.0000000849 (PMC12889919; doi:10.1681/ASN.0000000849)
Supplement: Supplementary file 1 [file jasn-37-299-s001.pdf]

ASN American Society of Nephrology | JASN Journal of the American Society of Nephrology | KIDNEY360 Accessing Our World From Every Angle | CJASN Clinical Journal of the American Society of Nephrology

ASN Journal Disclosure Form

As per ASN journal policy, I have disclosed any financial relationships or commitments I have held in the past 36 months as included below. I have listed my Current Employer below to indicate there is a relationship requiring disclosure. If no relationship exists, my Current Employer is not listed.

G. Chertow reports the following:
Employer: Stanford University School of Medicine; Consultancy: Akebia, Alebund, Ardelyx, AstraZeneca, CalciMedica, Miromatrix, Panoramic, Sanifit, Toku, Unicycive, Vertex; Ownership Interest: Ardelyx, CloudCath, Durect, Eliaz Therapeutics, Outset, Renibus, Unicycive; Research Funding: NIDDK, NIAID, CSL Behring; Advisory or Leadership Role: Board of Directors, Satellite Healthcare, Co-Editor, Brenner & Rector's The Kidney (Elsevier); and Other Interests or Relationships: DSMB service: NIDDK, George Institute, Aethlon, Bayer, Mineralys, ReCor.

I understand that the information above will be published within the journal article, if accepted, and that failure to comply and/or to accurately and completely report the potential financial conflicts of interest could lead to the following: 1) Prior to publication, article rejection, or 2) Post-publication, sanctions ranging from, but not limited to, issuing a correction, reporting the inaccurate information to the authors' institution, banning authors from submitting work to ASN journals for varying lengths of time, and/or retraction of the published work.

Name: Glenn M. Chertow
Manuscript ID: JASN-2025-000889R1
Manuscript Title: Efficacy and safety of baxdrostat in participants with chronic kidney disease and uncontrolled hypertension (FigHTN)
Date of Completion: July 30, 2025
Disclosure Updated Date: January 22, 2025

ASN American Society of Nephrology · JASN Journal of the American Society of Nephrology · KIDNEY360 Accessing Our World From Every Angle · CJASN Clinical Journal of the American Society of Nephrology

ASN Journal Disclosure Form

As per ASN journal policy, I have disclosed any financial relationships or commitments I have held in the past 36 months as included below. I have listed my Current Employer below to indicate there is a relationship requiring disclosure. If no relationship exists, my Current Employer is not listed.

J. Dwyer reports the following:
Employer: University of Utah Health; Consultancy: Acelyrin Acuta Capital Akebia Alnylam Altimmune Ardelyx Arrowhead; AZ Bayer Biogen; BioRasi BioVie; BD; BI Cincor CinRx CSL Therapeutics Fibrogen Fortrea; GSK; Icon; Intercept Pharmaceuticals; Inversago Ionis Ipsen Biosciences JucaBio KardiganBio, LabCorp Lilly Medpace MicuRX Novo Nordisk Novotech ProKidney Recor Medical, RenalytixAI RRD International Transcend Therapeutics ValenzaBiotech Worldwide clinical trials; Ownership Interest: Venostent, Inc; PathEx, Inc; Acelyrin; Alumis; BioRasi, Inc; EphlaBio; Innovative Renal Care, LLC; Corventum, Inc; Teucer Biotech, Inc; Research Funding: CinCor; AstraZeneca; and Advisory or Leadership Role: Collaborative Study Group - Board of Directors and President;; The Bolles School - Board of Trustees.

I understand that the information above will be published within the journal article, if accepted, and that failure to comply and/or to accurately and completely report the potential financial conflicts of interest could lead to the following: 1) Prior to publication, article rejection, or 2) Post-publication, sanctions ranging from, but not limited to, issuing a correction, reporting the inaccurate information to the authors' institution, banning authors from submitting work to ASN journals for varying lengths of time, and/or retraction of the published work.

Name: Jamie P. Dwyer
Manuscript ID: JASN-2025-000889
Manuscript Title: Efficacy and safety of baxdrostat in participants with chronic kidney disease and uncontrolled hypertension (FigHTN)
Date of Completion: July 9, 2025
Disclosure Updated Date: July 9, 2025

ASN
American Society of Nephrology
JASN
Journal of the American Society of Nephrology
KIDNEY360
Accessing Our World From Every Angle
CJASN
Clinical Journal of the American Society of Nephrology

ASN Journal Disclosure Form

As per ASN journal policy, I have disclosed any financial relationships or commitments I have held in the past 36 months as included below. I have listed my Current Employer below to indicate there is a relationship requiring disclosure. If no relationship exists, my Current Employer is not listed.

H. Heerspink reports the following:
Employer: University Medical Center Groningen; Consultancy: Ongoing consultancy agreements with AstraZeneca, Alexion, Bayer, Boehringer Ingelheim, Biocity Therapeutic, Dimerix, Eli-Lilly, Gilead, Janssen, Novartis, NovoNordisk, Roche, Travere Therapeutics; Research Funding: AstraZeneca, Bayer, Boehringer Ingelheim, NovoNordisk and Janssen research support (grant funding directed to employer); Honoraria: Lecture fees from AstraZeneca and NovoNordisk; and Speakers Bureau: AstraZeneca, Bayer, Novo Nordisk.

I understand that the information above will be published within the journal article, if accepted, and that failure to comply and/or to accurately and completely report the potential financial conflicts of interest could lead to the following: 1) Prior to publication, article rejection, or 2) Post-publication, sanctions ranging from, but not limited to, issuing a correction, reporting the inaccurate information to the authors' institution, banning authors from submitting work to ASN journals for varying lengths of time, and/or retraction of the published work.

Name: Hiddo Jan L. Heerspink
Manuscript ID: JASN-2025-000889
Manuscript Title: Efficacy and safety of baxdrostat in participants with chronic kidney disease and uncontrolled hypertension (FigHTN)
Date of Completion: July 8, 2025
Disclosure Updated Date: June 7, 2025

ASN American Society of Nephrology JASN Journal of the American Society of Nephrology KIDNEY360 Accessing Our World From Every Angle CJASN Clinical Journal of the American Society of Nephrology

ASN Journal Disclosure Form

As per ASN journal policy, I have disclosed any financial relationships or commitments I have held in the past 36 months as included below. I have listed my Current Employer below to indicate there is a relationship requiring disclosure. If no relationship exists, my Current Employer is not listed.

D. Little reports the following:
Employer: AstraZeneca; Ownership Interest: AstraZeneca; Research Funding: I work for AstraZeneca and as such I have participated in research sponsored by AstraZeneca; and Other Interests or Relationships: I volunteer as a nephrologist at Walter Reed National Military Medical Center.

I understand that the information above will be published within the journal article, if accepted, and that failure to comply and/or to accurately and completely report the potential financial conflicts of interest could lead to the following: 1) Prior to publication, article rejection, or 2) Post-publication, sanctions ranging from, but not limited to, issuing a correction, reporting the inaccurate information to the authors' institution, banning authors from submitting work to ASN journals for varying lengths of time, and/or retraction of the published work.

Name: Dustin J. Little
Manuscript ID: JASN-2025-000889
Manuscript Title: Efficacy and safety of baxdrostat in participants with chronic kidney disease and uncontrolled hypertension (FigHTN)
Date of Completion: July 28, 2025
Disclosure Updated Date: June 6, 2025

ASN American Society of Nephrology
JASN Journal of the American Society of Nephrology
KIDNEY360 Accessing Our World From Every Angle
CJASN Clinical Journal of the American Society of Nephrology

ASN Journal Disclosure Form

As per ASN journal policy, I have disclosed any financial relationships or commitments I have held in the past 36 months as included below. I have listed my Current Employer below to indicate there is a relationship requiring disclosure. If no relationship exists, my Current Employer is not listed.

N. Maklad reports the following:
Employer: Astrazeneca

I understand that the information above will be published within the journal article, if accepted, and that failure to comply and/or to accurately and completely report the potential financial conflicts of interest could lead to the following: 1) Prior to publication, article rejection, or 2) Post-publication, sanctions ranging from, but not limited to, issuing a correction, reporting the inaccurate information to the authors' institution, banning authors from submitting work to ASN journals for varying lengths of time, and/or retraction of the published work.

Name: Noha S. Maklad
Manuscript ID: JASN-2025-000889
Manuscript Title: Efficacy and safety of baxdrostat in participants with chronic kidney disease and uncontrolled hypertension (FigHTN)
Date of Completion: July 14, 2025
Disclosure Updated Date: July 14, 2025

ASN American Society of Nephrology JASN Journal of the American Society of Nephrology KIDNEY360 Accessing Our World From Every Angle CJASN Clinical Journal of the American Society of Nephrology

ASN Journal Disclosure Form

As per ASN journal policy, I have disclosed any financial relationships or commitments I have held in the past 36 months as included below. I have listed my Current Employer below to indicate there is a relationship requiring disclosure. If no relationship exists, my Current Employer is not listed.

J. Monyak reports the following:
Employer: AstraZeneca; and Ownership Interest: AstraZeneca.

I understand that the information above will be published within the journal article, if accepted, and that failure to comply and/or to accurately and completely report the potential financial conflicts of interest could lead to the following: 1) Prior to publication, article rejection, or 2) Post-publication, sanctions ranging from, but not limited to, issuing a correction, reporting the inaccurate information to the authors' institution, banning authors from submitting work to ASN journals for varying lengths of time, and/or retraction of the published work.

Name: John Monyak
Manuscript ID: JASN-2025-000889
Manuscript Title: Efficacy and safety of baxdrostat in participants with chronic kidney disease and uncontrolled hypertension (FigHTN)
Date of Completion: July 14, 2025
Disclosure Updated Date: July 14, 2025

ASN American Society of Nephrology JASN Journal of the American Society of Nephrology KIDNEY360 Accessing Our World From Every Angle CJASN Clinical Journal of the American Society of Nephrology

ASN Journal Disclosure Form

As per ASN journal policy, I have disclosed any financial relationships or commitments I have held in the past 36 months as included below. I have listed my Current Employer below to indicate there is a relationship requiring disclosure. If no relationship exists, my Current Employer is not listed.

R. Myte reports the following:
Employer: AstraZeneca; and Other Interests or Relationships: Current employee and stockholder of AstraZeneca. ;.

I understand that the information above will be published within the journal article, if accepted, and that failure to comply and/or to accurately and completely report the potential financial conflicts of interest could lead to the following: 1) Prior to publication, article rejection, or 2) Post-publication, sanctions ranging from, but not limited to, issuing a correction, reporting the inaccurate information to the authors' institution, banning authors from submitting work to ASN journals for varying lengths of time, and/or retraction of the published work.

Name: Robin Myte
Manuscript ID: JASN-2025-000889
Manuscript Title: Efficacy and safety of baxdrostat in participants with chronic kidney disease and uncontrolled hypertension (FigHTN)
Date of Completion: July 8, 2025
Disclosure Updated Date: July 8, 2025

ASN
American Society of Nephrology
JASN
Journal of the American Society of Nephrology
KIDNEY360
Accessing Our World From Every Angle
CJASN
Clinical Journal of the American Society of Nephrology

ASN Journal Disclosure Form

As per ASN journal policy, I have disclosed any financial relationships or commitments I have held in the past 36 months as included below. I have listed my Current Employer below to indicate there is a relationship requiring disclosure. If no relationship exists, my Current Employer is not listed.

O. Vedin reports the following:
Employer: AstraZeneca; and Ownership Interest: AstraZeneca pension fund.

I understand that the information above will be published within the journal article, if accepted, and that failure to comply and/or to accurately and completely report the potential financial conflicts of interest could lead to the following: 1) Prior to publication, article rejection, or 2) Post-publication, sanctions ranging from, but not limited to, issuing a correction, reporting the inaccurate information to the authors' institution, banning authors from submitting work to ASN journals for varying lengths of time, and/or retraction of the published work.

Name: Ola Vedin
Manuscript ID: JASN-2025-000889
Manuscript Title: Efficacy and safety of baxdrostat in participants with chronic kidney disease and uncontrolled hypertension (FigHTN)
Date of Completion: July 7, 2025
Disclosure Updated Date: July 7, 2025
