# Supplementary material for: Efficacy and Safety of Baxdrostat in Participants with CKD and Uncontrolled Hypertension: A Randomized, Double-Blind, Placebo-Controlled Trial
Source: J Am Soc Nephrol. 2025 Sep 6;37(2):299–311. doi: 10.1681/ASN.0000000849 (PMC12889919; doi:10.1681/ASN.0000000849)
Supplement: Supplementary file 2 [file jasn-37-299-s002.pdf]

Supplemental Material

Supplemental Methods

Supplemental Figure 1. Least square means (95% CI) change from baseline to Week 26 in eGFR over time (exploratory outcome) in the A) pooled baxdrostat treatment group and B) low- and high-dose baxdrostat groups compared with placebo

Supplemental Table 1. Least square mean change from baseline to Week 26 in seated systolic BP for participants receiving baxdrostat (pooled) vs. placebo stratified by SGLT2 inhibitor use and eGFR (mITT population; exploratory sub-analysis)

Supplemental Table 2. Exploratory outcomes

Supplemental Table 3. Percentage change from baseline to Week 26 in geometric mean serum aldosterone and serum renin over time in the low- and high-dose baxdrostat groups compared with placebo (exploratory PD sub-analysis)

Supplemental Table 4. Incidence of treatment-emergent serum potassium abnormalities (safety outcome)

Statistical Analysis Plan

Supplemental Methods

Contents:

1. Inclusion criteria
2. Exclusion criteria
3. Racial and ethnicity reporting
4. Guidelines for titration
5. Description of clinical and laboratory measurements
6. Additional statistical analyses information
7. Supplementary Figures
8. Supplementary Tables

Inclusion criteria:

Patients who met all of the following criteria were eligible to participate in the study:

1. Adult male or female participant ≥18 years of age.
2. Mean seated office systolic blood pressure (BP) ≥140 mmHg at screening, run-in period, and Week 0.
    a. Participants with mean seated office systolic BP ≥130 mmHg may have been eligible if they had diabetes.
    b. Mean seated systolic BP was defined as the average of three seated office systolic BP measurements at any single clinical site visit.
3. Had a prior diagnosis of mild-to-severe CKD, defined as eGFR (based on the CKD-EPI equation) of 25 to 75 mL/min/1.73 m^2, inclusive, at screening.

    a. To ensure participants with moderate and severe renal impairment were represented, the number of participants with an eGFR ≥60 and <75 mL/min/1.73 m^2 was capped at 45 participants.

4. Had a urine albumin-creatinine ratio (UACR) ≥100 mg/g (≥11.3 mg/mmol) in at least two out of three measurements based on first urine collected in the morning on consecutive days during the screening period.
5. Was taking an angiotensin-converting enzyme inhibitor (ACEi) or angiotensin receptor blockers (ARB) at the participant's maximum tolerated daily dose, based on investigator judgment, for >4 weeks prior to screening.
6. If taking a sodium-glucose co-transporter 2 (SGLT2) inhibitor at screening, the regimen must have been stable for a period of at least 8 weeks before screening and was expected to remain at a stable dose over the study period.
    a. It was expected that participants currently taking an SGLT2 inhibitor at screening would not initiate this class of medication during the entire study period.
7. Was willing to be compliant with the contraception and reproductive restrictions of the study as follows:
    a. Female participants of childbearing potential (i.e., ovulating, premenopausal, and not surgically sterile) must have had a documented negative pregnancy test at screening and the randomization visit (Week 0); and must have used a highly effective method of contraception (i.e., <1% failure rate) from Day 1 through 30 days after the last administration of study drug.
        i. Acceptable methods of contraception for female participants of childbearing potential enrolled in the study included the following:
            1. Surgical sterilization (tubal ligation)
            2. Intrauterine device for at least 12 weeks before screening

3. Hormonal contraception (oral, implant, injection, ring, or patch) for at least 12 weeks before screening
4. Diaphragm used in combination with spermicide

b. Postmenopausal women must have not had menstrual bleeding for at least 1 year before initial dosing and were either >60 years or had an elevated follicle-stimulating hormone (FSH) level >40 mIU/mL at screening.

8. Was able and willing to give informed consent for participation in the study.
9. After the mandatory run-in period of 2 weeks, investigators must have confirmed the participant's blood pressure (BP) and UACR measurements still met the eligibility criteria.

Exclusion criteria:

Participants who met any of the following criteria were excluded from participation in the study:

1. Had a documented diagnosis of type 1 diabetes.
2. Were not willing or not able to discontinue a mineralocorticoid receptor agonist (MRA) or a potassium-sparing diuretic as part of an existing antihypertensive regimen.
   a. Participants taking an MRA or a potassium-sparing diuretic (e.g., triamterene, amiloride, etc.) as an antihypertensive agent must have been willing to discontinue this agent for study eligibility. The potassium-sparing diuretic may have been discontinued and replaced with a non-potassium-sparing diuretic. All participants who remained on a stable regimen of antihypertensive agents, including a non-potassium-sparing diuretic, for at least 6 weeks, were eligible to enter the single-blind run-in period. If the participant discontinued their prior MRA or potassium-sparing diuretic and/or initiated a new antihypertensive for

study eligibility or had their antihypertensive dose adjusted after screening, they remained on a stable regimen of antihypertensive agents for at least 4 weeks and had an extended screening period of up to 9 weeks from signing of informed consent to randomization (Week 0).

3. Had a single occurrence of mean seated office systolic BP >180 mmHg or diastolic BP >110 mmHg during the screening period (if such a BP was recorded during the screening period, the participant may have attended an interim visit for an additional BP measurement and reassessment of inclusion/exclusion criteria).
    a. Mean seated office BP was defined as the average of three measurements obtained at any one clinical site visit. If the participant missed the regularly scheduled antihypertensive medication(s) prior to screening, one BP retest was allowed ≥2 hours after taking the medication(s), on the following day, or later after reestablishing the regularly scheduled antihypertensive regimen.
4. Had a body mass index (BMI) >50 kg/m^2 at screening.
5. Had documented bilateral clinically relevant renal artery stenosis of ≥70%; if the imaging evidence was met, the participant was excluded, since hypertension itself could be considered ‘clinically relevant’. Suspected or nondocumented renal artery stenosis was not excluded.
6. Had dialysis for acute kidney injury/acute renal failure within 12 weeks prior to the screening period or had a planned dialysis or kidney transplantation during the course of the study.
7. Had known documented chronic heart failure New York Heart Association Class III or Class IV and/or hospitalization for heart failure within 6 months of screening.
8. Had a stroke, transient ischemic attack, hypertensive encephalopathy, acute coronary syndrome, or hospitalization for heart failure within 6 months of screening.

9. Had known current severe left ventricular outflow obstruction, such as obstructive hypertrophic cardiomyopathy and/or severe aortic valvular disease diagnosed from a prior echocardiogram or another imaging study.
10. Had a planned coronary revascularization (percutaneous coronary intervention [PCI] or coronary artery bypass graft [CABG]) or any major surgical procedure during the study.
11. Had PCI, CABG, other major cardiac surgery (e.g., valve replacement), or peripheral arterial bypass surgery within 6 months of screening.
12. Had a prior solid organ transplant or cell transplant.
13. Was expected to receive or was receiving any of the exclusionary drugs, such as strong inducers of cytochrome P450 (CYP) 3A, chronic (medication was taken more than three times a week for more than 3 months) use of non-steroidal anti-inflammatory drugs, spironolactone/eplerenone, and/or chronic use of systemic steroids.
14. Had a known hypersensitivity to any of the following:
    a. CIN-107 or drugs of the same class
    b. Excipients in CIN-107 or drugs of the same classes
15. Had received immunotherapy for treatment of CKD within 6 months of Visit 1 or expected to receive immunotherapy for treatment of CKD during participation in the study.
16. Had any clinically relevant medical or surgical conditions including unstable conditions and/or conditions requiring regular transfusion or treatment with systemic immunosuppressants, including corticosteroids that, in the opinion of the investigator, would have put the participant at risk by participating in the study.
17. Had evidence of any of the following at screening (one retest was allowed):
    a. White blood cell count $>15x10^9/L$ or absolute neutrophil count $<1x10^9/L$
    b. Serum potassium <3.5 mEq/L

        i. Participants with a serum potassium level below normal range continued in the study without retest if the investigator elected to correct the serum potassium level with supplementation and offered to manage the condition.

    c. Serum potassium >5.0 mEq/L
    d. Serum sodium <135.0 mEq/L
    e. Serum aspartate aminotransferase or alanine aminotransferase >3 x upper limit of normal (ULN) or
    f. Total bilirubin >2 x ULN, unless due to Gilbert's syndrome
    g. eGFR <25 or >75 mL/min/1.73 m^2

18. Had uncontrolled diabetes with glycosylated hemoglobin >10.5% at screening.
19. Was positive for human immunodeficiency virus antibody, hepatitis B surface antigen, or hepatitis C virus ribonucleic acid.
20. Had typical consumption of >14 alcoholic drinks weekly.
    a. One drink of alcohol is equivalent to a half-pint of beer (285 mL), one glass of spirits (25 mL), or one glass of wine (125 mL).
21. Had participated in another clinical study involving any investigational drug within 30 days prior to screening or planned to participate in another clinical study within 30 days of discontinuation of study drug.
22. Had received experimental therapy for disease intervention with a small molecule within 30 days of screening or 5 half-lives, whichever was longer, or received experimental therapy with a large molecule within 90 days of screening or 5 half-lives, whichever was longer.
    a. Vaccinations, including those for coronavirus disease 2019 (COVID-19), were not exclusionary.
23. Was pregnant, breastfeeding, or planned to become pregnant during the study.

24. Was considered by the investigator, after reviewing medical and psychiatric history, physical examination, and laboratory evaluations, to be unsuitable for any other reason that may have either placed the participant at increased risk during participation or interfered with the interpretation of the study outcomes. History of COVID-19 infection, in itself, was not an exclusionary criterion unless the participant was subsequently considered unsuitable for the study based on criteria above.

Racial and ethnicity reporting:

Participant demographics were self-reported. Racial demographics were reported according to FDA guidelines. Ethnicity was reported as 'Hispanic or Latino' and race as American Indian or Alaska Native, Asian, Black or African American, Native Hawaiian or Other Pacific Islander, White or Multiple. Demographic data were collected in order to accurately represent participants from racial groups across the US and to ensure external validity.

Guidelines for Titration:

Participants randomized to the low-dose group initially received 0.5 mg baxdrostat; participants in the high-dose group initially received 2 mg baxdrostat. At Week 3, the baxdrostat dose could be up-titrated to 1 mg for the low-dose group, and to 4 mg for the high-dose group. To maintain the blind, placebo was titrated using the same rules, and – because the 4 mg dose required two baxdrostat tablets – all participants took two study drug tablets throughout the study.

Description of clinical and laboratory measurements

From Week 0 to Week 28, BP and blood laboratory parameters (serum potassium and sodium, standard safety chemistry panel, hematology, coagulation, and urinalysis) were measured at every visit (Weeks 1, 3, 4, 6, 9, 16, 22, 26, and 28); UACR was measured at Week 16 and 26, using three consecutive first-morning void samples collected by

participants at home. Seated BP was measured in triplicate with automated office blood pressure monitoring (AOBPM). Diastolic BP was measured by AOBPM.

Additional statistical analyses information

The sample size for the study, assuming an early withdrawal rate of 8%, was calculated to include approximately 174 participants (approximately 58 participants each in the low-dose, high-dose, and placebo groups), sufficient for 90% power for the primary endpoint assuming a treatment difference of 6 (standard deviation [SD] 11) mmHg to detect a difference between the pooled baxdrostat and placebo groups using a two-sided significance level of 0.05. All statistical analyses were performed using SAS® version 9.4 or higher.

Demographics and baseline characteristics were summarized using descriptive statistics. Categorical data were summarized in terms of the number of participants providing data at the relevant time point, frequency counts, and percentages. For efficacy and safety endpoints, baseline was defined as the last non-missing value obtained prior to the first dose/administration of baxdrostat, unless otherwise specified.

Participants were randomized using a computer-generated, stratified, randomized block list (with 3 stratification factors and blocking within each stratum for a block size of 6) implemented via an automated interactive response technology (IRT) system. Medidata Solutions generated the randomization list. The list was incorporated into the interactive response technology (IRT) system, and the IRT system was accessed by investigators who used it to enroll patients. The automated IRT system implemented the randomization list by sequentially allocating randomized participants to the next treatment in the list (within the stratum the patient qualified). Once randomized, the IRT system assigned participants to the intervention.

Supplemental Figure 1. Least square means (95% CI) change from baseline to Week 26 in eGFR over time (exploratory outcome) in the A) pooled baxdrostat treatment group and B) low- and high-dose baxdrostat groups compared with placebo

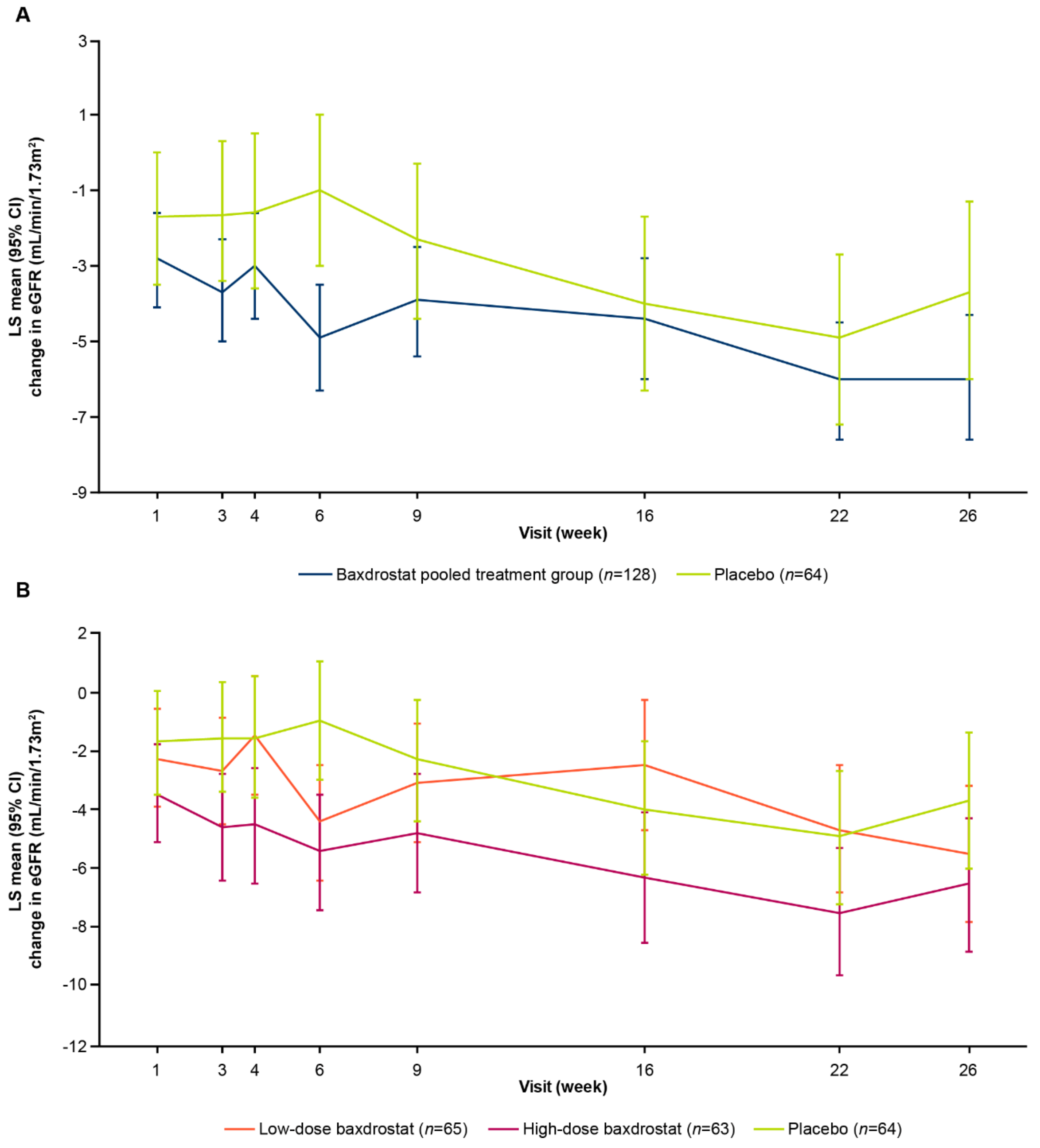

Least square means for baxdrostat pooled group and placebo are from a MMRM including fixed effects for treatments (baxdrostat treatment groups vs. placebo), visit, stratification variables (SGLT2

inhibitor use and systolic BP category), and the treatment-by-visit interaction, along with a covariate of the baseline eGFR value and the baseline eGFR-by-visit interaction.

BP, blood pressure; CI, confidence interval; eGFR, estimated glomerular filtration rate; MMRM, mixed-effect model for repeated measures; SGLT2, sodium-glucose cotransporter 2.

Supplemental Table 1. Least square mean change from baseline to Week 26 in seated systolic BP for participants receiving baxdrostat (pooled) vs. placebo stratified by SGLT2 inhibitor use and eGFR (mITT population; exploratory sub-analysis)

	Treatment group	N	n	LS mean (SE)	LS mean difference (95% CI)
**Baseline SGLT2 inhibitor use**					
**Yes**	Baxdrostat pooled	42	36	–14.2 (2.6)	–5.5 (–14.7, 3.7)
	Placebo	21	16	–8.8 (3.9)	
**No**	Baxdrostat pooled	86	69	–15.9 (1.9)	–9.4 (–15.9, –2.9)
	Placebo	43	32	–6.5 (2.7)	
**Baseline eGFR**					
**<45 mL/min/1.73m^2**	Baxdrostat pooled	69	57	–14.6 (2.1)	–10.1 (–17.3, –2.9)
	Placebo	34	26	–4.5 (3.0)	
**≥45 mL/min/1.73m^2**	Baxdrostat pooled	59	48	–16.2 (2.3)	–5.9 (–13.7, 1.8)
	Placebo	30	22	–10.2 (3.3)	

The analysis was performed using a MMRM including fixed effects for treatment (baxdrostat treatment groups pooled vs placebo), visit, stratification variable eGFR category/ SGLT2 inhibitor use, baseline SGLT2 inhibitor /eGFR category, treatment-by-visit interaction, baseline SGLT2 inhibitor/eGFR category-by-visit interaction, baseline SGLT2 inhibitor/ eGFR category-by-treatment interaction and baseline SGLT2 inhibitor/eGFR category-by-visit by-treatment interaction, along with a covariate of the baseline seated systolic BP value and the baseline seated systolic BP by visit interaction.

BP, blood pressure; CI, confidence interval; eGFR, estimated glomerular filtration rate; LS, least square; mITT, modified intent-to-treat; MMRM, mixed-effect model for repeated measures; n, number

of patients per category; N, number of patients per treatment group; SE, standard error; SGLT2 inhibitor, sodium-glucose cotransporter-2 inhibitor**.**

Supplemental Table 2. Exploratory outcomes

	**Baxdrostat low-dose** *n*=65	**Baxdrostat high-dose** *n*=63	**Baxdrostat treatment groups pooled** *n*=128	**Placebo** *n*=64
Number of participants in BP analyses	50	55	105	48
Achieved seated systolic BP <130 mmHg at Week 26, n/N (%)	21/50 (42)	17/55 (31)	38/105 (36)	8/48 (17)
Change from baseline to Week 26 in seated diastolic BP, mean (SD) mmHg	–6.5 (11.4)	–4.9 (8.9)	–5.6 (10.2)	–1.0 (8.4)
LS mean (95% CI) difference in diastolic BP vs. placebo	–5.0 (–8.6, –1.5)	–4.7 (–8.2, –1.1)	–4.8 (–7.9, –1.8)	
Number of participants in UACR analysis	49	54	103	47
Percent change from baseline to Week 26 in geometric mean UACR (95% CI) compared with placebo	–52.0 (–66.8, –30.6)	–58.0 (–70.8, –39.4)	–55.2 (–67.4, –38.3)	
Number of participants in eGFR analysis	50	55	105	49
LS mean difference (95% CI) in eGFR from baseline to Week 26 compared to placebo, mL/min/1.73 m^2	–1.8 (–5.0, 1.4)	–2.8 (–6.0, 0.4)	–2.3 (–5.1, 0.5)	
Proportion of participants who experienced >30% decline in eGFR from one visit to the next, n/N (%)	10/65 (15)	9/63 (14)	19/128 (15)	11/64 (17)
Proportion of participants who experienced >50% decline in eGFR from one visit to the next, n/N (%)	0	0	0	1/64 (1.6)

BP, blood pressure; CI, confidence interval; eGFR, estimated glomerular filtration rate; LS, least square; n, number of patients in the analysis; N, number of patients per treatment group; SD, standard deviation; UACR, urine albumin-to-creatinine ratio.

Supplemental Table 3. Percentage change from baseline to Week 26 in geometric mean serum aldosterone and serum renin over time in the low- and high-dose baxdrostat groups compared with placebo (exploratory PD sub-analysis)

PD parameter (unit)	Treatment group	N	n	Percentage change from baseline (95% CI)	Percentage change from baseline compared to placebo (95% CI)
**Aldosterone (ng/dL)**	Baxdrostat high-dose	63	54	–61.9 (–68.1, –54.4)	–60.1 (–69.1, –48.5)
	Baxdrostat low-dose	65	48	–61.4 (–68.1, –53.4)	–59.7 (–68.9, –47.7)
	Placebo	63	45	–4.4 (–21.2, 16.0)	
**Renin (ng/L)**	Baxdrostat high-dose	63	45	141.3 (71.9, 238.7)	78.8 (10.4, 189.8)
	Baxdrostat low-dose	65	43	86.6 (32.6, 162.7)	38.3 (–14.4, 123.5)
	Placebo	63	37	34.9 (–6.3, 94.3)	

Analyses for each PD parameter were performed using MMRMs similar to the UACR analysis in Figure 4. LS mean estimates were back-transformed to calculate GMR and percentage change from baseline was calculated as (GMR-1) × 100. All patients who received at least 1 dose of any study drug and had at least one quantifiable PD parameter value were included in the analysis.

CI, confidence interval; GMR, geometric mean ratio; LS, least square; MMRM, mixed-effect model for repeated measures; n, number of patients in the analysis; N, number of patients per treatment group; PD, pharmacodynamic; UACR, urine albumin-to-creatinine ratio.

Supplemental Table 4. Incidence of treatment-emergent serum potassium abnormalities (safety outcome)

Serum potassium (mmol/L), n (%)[a]	**Baxdrostat low-dose** ***n*****=65**	**Baxdrostat high-dose** ***n*****=63**	**Baxdrostat treatment groups pooled** ***n*****=128**	**Placebo** ***n*****=64**	**Total** ***N*****=192**
>5.5 mmol/L	23 (35.4)	24 (38.1)	47 (36.7)	3 (4.7)	50 (26.0)
Confirmed >5.5 mmol/L	11 (16.9)	9 (14.3)	20 (15.6)	0	20 (10.4)
>6.0 mmol/L	9 (13.8)	5 (7.9)	14 (10.9)	0	14 (7.3)
Confirmed >6.0 mmol/L	2 (3.1)	0	2 (1.6)	0	2 (1.0)
≥5.5 mmol/L	25 (38.5)	28 (44.4)	53 (41.4)	6 (9.4)	59 (30.7)
Confirmed ≥5.5 mmol/L	13 (20.0)	12 (19.0)	25 (19.5)	0	25 (13.0)
≥6.0 mmol/L	10 (15.4)	12 (19.0)	22 (17.2)	2 (3.1)	24 (12.5)
Confirmed ≥6.0 mmol/L	3 (4.6)	2 (3.2)	5 (3.9)	0	5 (2.6)

[a] Participants with multiple events in the same category are counted only once in that category.

A treatment emergent abnormality is defined as an abnormal laboratory result according to the criteria at any post-baseline (scheduled or unscheduled) visit. A confirmed abnormality is defined as a treatment-emergent abnormality that is verified by a second test showing the same abnormality in the first consecutive measurement after the initial test.

AstraZeneca AB

D6972C00001/ FigHtn-CKD CIN-107-123 Number

A Randomized, Double-Blind, Placebo-Controlled, Multicenter, Parallel-Group, Dose-Ranging Study to Evaluate CIN-107 for the Treatment of Patients with Uncontrolled Hypertension and Chronic Kidney Disease

Statistical Analysis Plan

Version: 2.0

Parexel Project Number: 271586

Sponsor Signature Page

This document has been approved and signed electronically on the final page by the following:

Approved by:	____________________	____________
	Name	Date

PAREXEL Signature Page

This document has been approved and signed electronically on the final page by the following:

Approved by: ____________________________ ________________

Name Date

Table of Contents

Sponsor Signature Page **2**

PAREXEL Signature Page **3**

Table of Contents **4**

Version History **6**

1. INTRODUCTION **9**

2. STUDY OBJECTIVES, ENDPOINTS, AND ESTIMANDS **10**
2.1. Study Objectives and Endpoints 10
2.2. Estimands 12
2.2.1. Primary Estimands 12
2.2.2. Secondary and Exploratory Estimands 13

3. INVESTIGATIONAL PLAN **14**
3.1. Study Design 14
3.1.1. Treatment Assignments 15
3.1.2. Planned Analyses 16
3.2. Statistical Hypothesis 16
3.2.1. Primary Efficacy Analysis 16
3.2.2. Secondary Efficacy Analysis 16
3.2.3. Exploratory Analysis 18
3.2.4. PK Analysis 19
3.2.5. PD Analysis 19

4. STATISTICAL METHODS **21**
4.1. Data Quality Assurance 21
4.2. General Presentation Considerations 21
4.3. General variables 22
4.3.1. Baseline 22
4.3.2. Relative Day 23
4.3.3. Visit Windows 23
4.3.4. Handling Missing Data 24
4.4. Software 28
4.5. Study Patients 28
4.5.1. Disposition of Patients 28
4.5.2. Protocol Deviations 29
4.5.3. Analysis Sets 29
4.5.4. Demographics and Baseline Characteristics 30
4.5.5. Medical History 30
4.5.6. Surgical History 30
4.5.7. Prior and Concomitant Medication 31
4.6. Primary Efficacy Analysis 31
4.6.1. Definition of Endpoint(s) 31
4.6.2. Main Analytical Approach 32

4.7. Secondary Efficacy Analysis 33
4.7.1. Definition of Endpoint(s) 33
4.7.2. Main Analytical Approach 33
4.8. Exploratory Endpoints 33
4.8.1. Percentage of Patients Achieving SBP < 130 mmHg at Week 36 in CIN-107 Treatment Groups 34
4.8.2. Change from baseline in DBP measured by AOBPM with each dosing strategy of CIN-107 compared to placebo at Week 26. 34
4.8.3. The Percentage of Change from Baseline in UACR with Each Dosing Strategy of CIN-107 Compared to Placebo at Week 26. 35
4.8.4. Change from Baseline in eGFR at Week 26 35
4.8.5. To Determine the Changes from Baseline in the PD Variables of Each Dose Strength of CIN-107 Compared to Placebo at Week 26 36
4.8.6. To Evaluate the Dose-response Relationships of CIN-107 in Patients with uHTN and CKD Using Measures of Safety, PD, and/or Efficacy at Week 26 37
4.9. Sensitivity 37
4.10. Safety Analyses 38
4.10.1. Extent of Exposure 38
4.10.2. Study Drug Compliance 39
4.10.3. Adverse Events 40
4.10.4. Clinical Laboratory Evaluation 43
4.10.5. Physical Examination findings 47
4.10.6. Vital Signs 47
4.10.7. ECG Results 48
4.10.8. Pharmacokinetics 48
4.10.9. Pharmacodynamics 50
4.10.10. Pregnancy Test 51
4.11. Interim Analysis 51
4.12. Data Safety Monitoring Board 51
4.13. Unblinded Review Committee 52
4.14. Changes to Protocol-planned Analyses 52
4.15. Sample Size Determination 52
5. SUPPORTING DOCUMENTATION 53
Appendix 1: List of Abbreviations 53
6. REFERENCES 56

Version History

SAP Version	Date	Change	Rationale
1.0	31 Jan 2023	Not applicable.	Original version.
2.0	TBD	Updated study protocol version and eCRF version numbers and dates.	New documents since finalization of the SAP.
		Updated the study objectives and endpoints.	To comply with the protocol amendment (CSP Version 4.0 dated 17 Aug 2023).
		Updated analysis set.	To comply with AZ updated definitions for analyses set.
		Added a section 3.2 Statistical Hypothesis	To elaborate the hypothesis tested as primary and secondary efficacy endpoints
		Added figure	To explain the hierarchical test procedure for the primary and efficacy endpoints
		Added subgroup analysis for primary and secondary endpoints in section 3.2.2.	sponsor decision
		Added the subgroup analysis for exploratory end point UACR in section 3.2.3	sponsor decision
		Added sections 3.2.4 PK Analysis and 3.2.5 PD Analysis	Sponsor decision
		Elaborated general presentation consideration section 4.2	To make sure all relevant presentation requirements are covered

		Baseline for SBP, DBP, UACR, effiacy and safety variables were explained in detail in section 4.3.1	To cover different baseline considerations for different variables
		Table 2 Visit windowing was revised	sponsor decision
		Revised Table 3: Analysis sets	As per protocol amendment
		Section 4.5.6 Surgical history added	Sponsor decision
		Added sections 4.6, 4.7 and 4.8	To elaborate the exploratory endpoint analysis
		In section 4.9, added a second sensitiviy analaysis of primary endpoint if data permits: excluding the data prior to an unblinding event happened in fall of	Sponsor request
		Revised section 4.10.3 adverse events by listing all AE, TEAE summaries	Sponsor request
		Added defenitions of All AEs, TEAEs on-treatment and TEAEs of on-study in section 4.10.3	Sponsor request
		Added Table 4 Laboratory variables and Table 5 vital signes pre-defined criteria.<br>Added a criteria that all quantitative laboratory values will be summarized in SI units except for UACR, which will be summarized in mg/g.<br><br>Revised the criteria for presenting lab abnormalities post baseline	For cross reference<br>Sponsor request<br><br>Sponsor request
		Added section 4.10.8 Pharmacokinetics presentation guidelines	previously missing
		Added section 4.10.9 Pharmacokinetics presentation guidelines.<br>Added new Tables – Table 6 Pharmacodynamic analytes – blood	previously missing and for cross reference

		sample and Table 7 Pharmacodynamic analytes – 24 hr urine sample	
		Removed the references	None of the references are cited within the body of SAP

1. INTRODUCTION

Aldosterone has been implicated in cardiovascular and kidney damage by causing inappropriate or excessive distal tubular reabsorption of sodium from the kidney. This can contribute to the development of hypertension and end-organ damage. Damage may also occur in permissive milieu with attendant high sodium intake, in which even normal concentrations of aldosterone produce blood pressure-independent end organ damage, acting through inflammatory and profibrotic pathways. Inappropriately high levels of aldosterone in patients with CKD have been suggested to contribute to CKD-associated hypertension by promoting inflammation, oxidative stress, fibrosis, mesangial cell proliferation, and podocyte injury. The blockage of aldosterone thereby represents a potential means to reduce BP as well as to mitigate end organ damage.

Baxdrostat (also known as CIN-107) is a highly potent, selective, and competitive inhibitor of aldosterone synthase and may be a novel treatment to provide added benefit for CKD patients when given in combination with an ACEi/ARB and/or other antihypertensive agents to reduce BP and improve renal function.

This SAP describes the detailed statistical methodology for the study CIN-107-123. Additionally, the SAP ensures that the data listings, tables, and figures will be complete and appropriate to reach valid conclusions regarding study objectives. The proposed statistical methodology in the SAP may also be included in the CSR, regulatory submissions, and future manuscripts.

The SAP complies with ICH Guideline E9 and is based on the following study documents:

- CSP, Version 4.0 (17 August 2023)
- eCRF Version 9.0 (27 November 2023)

2. STUDY OBJECTIVES, ENDPOINTS, AND ESTIMANDS

2.1. Study Objectives and Endpoints

Objectives	Endpoints
**Primary** [a]	
To evaluate the treatment effect of CIN-107 on SBP compared to placebo at Week 26 in patients with uHTN and CKD	Change in mean seated SBP from baseline to Week 26 in patients receiving CIN-107 compared to placebo [b]
**Secondary** [a]	
To evaluate the treatment effect of CIN-107 on SBP compared to placebo at Week 26 by dosing strategy	• The change from baseline of SBP in CIN-107 compared to placebo at Week 26 in patients assigned to the high-dose strategy group<br>• The change from baseline of SBP in CIN-107 compared to placebo at Week 26 in patients assigned to the low-dose strategy group
**Safety**	
To evaluate the safety and tolerability of CIN-107 from the time of randomization until the end of the follow-up period in patients with uHTN and CKD.<br>• To evaluate TEAEs<br>• To evaluate TE SAES<br>• To evaluate TE AESIs<br>• To evaluate TEAEs leading to premature discontinuation of study drug<br>• To evaluate treatment-emergent marked laboratory abnormalities<br>• To evaluate the change in standing SBP and DBP (measured pre-dose at the clinical site) from baseline to EOT (Visit 11)	• The incidence of TEAEs<br>• The incidence of TESAEs<br>• The incidence of TEAEs leading to premature discontinuation of the study drug<br>• The incidence of TE AESIs; AESIs will include the following:<br>• Hypotension events that require clinical intervention<br>• Abnormal potassium laboratory values that require clinical intervention<br>• Abnormal sodium laboratory values that require clinical intervention<br>• The incidence of TE marked laboratory abnormalities.<br>• Change in serum potassium and sodium levels from baseline to Week 26 between each dose strength of CIN-107 compared to placebo

Objectives	Endpoints
• To evaluate vital signs, standing BP, HR, physical examinations, ECGs, weight measurement, and clinical laboratory evaluations, including standard safety chemistry panel, hematology, coagulation, and urinalysis	• The change in standing SBP and DBP (measured at the clinical site prior to administration of study drug) from baseline to Week 26<br>• Vital signs (HR, respiratory rate, and body temperature), mean SBP, mean DBP, orthostatic vitals (standing BP and HR), physical examinations, ECG, weight measurement, and clinical laboratory evaluations including standard safety chemistry panel, hematology, coagulation, and urinalysis
**Exploratory** [a, c]	
To determine the percentage of patients achieving SBP < 130 mmHg at Week 26 by dosing strategy	• The percentage of patients achieving SBP < 130 mmHg at Week 26 in patients assigned to the high-dose strategy group<br>• The percentage of patients achieving SBP < 130 mmHg at Week 26 in patients assigned to the low-dose strategy group
To evaluate the change from baseline in DBP with each dosing strategy of CIN-107 compared to placebo at Week 26	• The change from baseline in DBP measured by AOBPM with each dosing strategy of CIN-107 compared to placebo at Week 26
To evaluate the change from baseline in urinary UACR with each dosing strategy of CIN-107 compared to placebo at Week 26	• The percentage of change from baseline in UACR with each dosing strategy of CIN-107 compared to placebo at Week 26
PD: To evaluate the dose-response relationships of CIN-107 in patients with uHTN and CKD using measures of safety, PD, and/or efficacy at Week 26<br>• To evaluate the relationship between change in UACR and changes in aldosterone and renin levels with each dose strength of CIN-107 compared to placebo<br>• To evaluate the relationship between change in UACR and changes in BP with each dose strength of CIN-107 compared to placebo<br>• To evaluate the relationship between the change in eGFR and changes in BP with each dose strength of CIN-107 compared to placebo<br>• To determine the changes from baseline in the PD variables of each dose strength of CIN-107 compared to placebo at Week 26	The PD endpoints are the changes from baseline over time with each dose strength of CIN-107 compared to placebo at Week 26 in PD variables including, but not limited to, the following:<br>• Serum and/or plasma parameters: electrolytes, aldosterone concentration, 11-deoxycorticosterone, corticosterone, 18 OH-corticosterone, cortisol, 11-deoxycortisol, NT-proBNP, PRA, direct renin concentration, angiotensinogen, angiotensin II, serum PICP, the ratio of PICP to CTX-1<br>• 24-hour urine parameters: electrolytes, aldosterone, albumin, creatinine, renin, MM-1, cystatin C, NGAL, protein<br>• The relationship between percentage of change in UACR and changes in aldosterone and renin

Objectives	Endpoints
	levels with each dose strength of CIN-107 compared to placebo at Week 26<br>• The relationship between change in UACR and changes in BP with each dose strength of CIN-107 compared to placebo at Week 26<br>• The relationship between the change in eGFR and changes in BP with each dose strength of CIN-107 compared to placebo at Week 26
PK/PD: To evaluate the exposure-response relationships of CIN-107 in patients with uHTN and CKD using measures of safety, PD, and/or efficacy [d]	• Percentage change from baseline in UACR at Week 26 in patients assigned to the high dose strategy group<br>• Percentage change from baseline in UACR at Week 26 in patients assigned to the low dose strategy group<br>• Change from baseline in eGFR at Week 26 in patients assigned to the high dose strategy group<br>• Change from baseline in eGFR at Week 26 of treatment in patients assigned to the low dose strategy group

[a] The mITT population will be used for the analysis of all primary and secondary efficacy endpoints.

[b] Estimand description and detailed intercurrent event strategies for the analysis of the primary efficacy endpoint are found in Section 2.2 and Table 1, respectively.

[c] Exploratory endpoints not part of the CSR are not included in the SAP. Those endpoints will be explained elsewhere.

[d] PK plasma concentration will be summarized and reported in CSR. The exposure response relationship of CIN-107 using measures of safety, PD and other efficacy parameters except UACR and eGFR will be explored outside the scope of the CSR. Hence it won't be included in the SAP.

2.2. Estimands

2.2.1. Primary Estimands

For the primary efficacy objective of evaluating the treatment effect of pooled CIN-107 in SBP compared to placebo at Week 26 in patients with uHTN and CKD, the estimand is defined as follows:

Treatment condition

- Placebo: placebo for CIN-107.

- Low-dosing CIN-107 strategy group: CIN-107 0.5mg and may up-titrate to take CIN-107 1 mg at Week 3 (Visit 5)
- High-dosing CIN-107 strategy group: CIN-107 2 mg and may up-titrate to take CIN-107 4 mg at Week 3 (Visit 5).

The dose strength may revert to the lower dose level in the patient's assigned group if the higher dose is not tolerated.

Target patient population: Adult male and female patients with uHTN and CKD. Endpoint: Change in seated SBP from baseline to Week 26.

Population-level summary: The treatment difference (pooled CIN-107-placebo) in the mean change from baseline at Week 26.

Intercurrent events and strategies: Intercurrent events include discontinuation of assigned study treatment for reasons unrelated to study drug, discontinuation of assigned study treatment for reasons related to study drug, receipt of other treatment for hypertension and CKD prior to Week 26, use of prohibited medication, and death. The strategies are described in**Table *1***.

Table 1: Intercurrent Event Strategies for the Primary Analysis of the Primary Efficacy Endpoint

Intercurrent Event	Strategy
Discontinuation of assigned study treatment	Treatment policy strategy: Primary efficacy endpoint data collected after treatment discontinuation will be included in the analysis.
Other treatment for hypertension received prior to Week 26	Treatment policy strategy: Primary efficacy endpoint data collected after use of other treatment for hypertension will be included in the analysis.
Use of prohibited medication	Treatment policy strategy: Primary efficacy endpoint data collected after use of prohibited medication will be included in the analysis.
Death	Hypothetical strategy: The hypothetical strategy is that the patient did not die. The primary efficacy endpoint will be considered missing.

2.2.2. Secondary and Exploratory Estimands

The same intercurrent events and strategies used for the primary efficacy endpoint will be used for secondary and exploratory efficacy endpoints.

3. INVESTIGATIONAL PLAN

3.1. Study Design

This is a phase II, randomized, double-blind, placebo-controlled, multicenter, parallel-group, dose ranging study to evaluate the efficacy and safety of CIN-107 for the treatment of hypertension in patients with uncontrolled hypertension (uHTN) and chronic kidney disease (CKD).

The patients who meet all inclusion and exclusion criteria will be randomized into one of the three treatment groups (High dosing strategy group, Low dosing strategy group and placebo) in 1:1:1 ratio.

The safety of CIN-107 will be assessed from the time of randomization until the end of the follow-up period. Patients will be followed for efficacy and adherence throughout the double-blind treatment period. Plasma, serum, and urine PD variables analyzed during the study will include measures of kidney function and aldosterone and its relevant precursors. Pharmacokinetic (PK) variables analyzed during the study will include plasma concentrations of CIN-107.

Patients will complete at least 12 visits over a period of approximately 8 months. The study consists of the following 3 periods and corresponding visits:

- A Screening Period of up to 4 weeks (Visit 1 and 2) including a 2-week Run-In Period (beginning at Visit 2 until Visit 3)
- A Double-Blind Treatment Period of 26 weeks (Visit 3 through 11)
- A Follow-Up Period of 2 weeks (Visit 12)

The study design schema is shown in Figure 1 study design.

Figure 1: Study Design

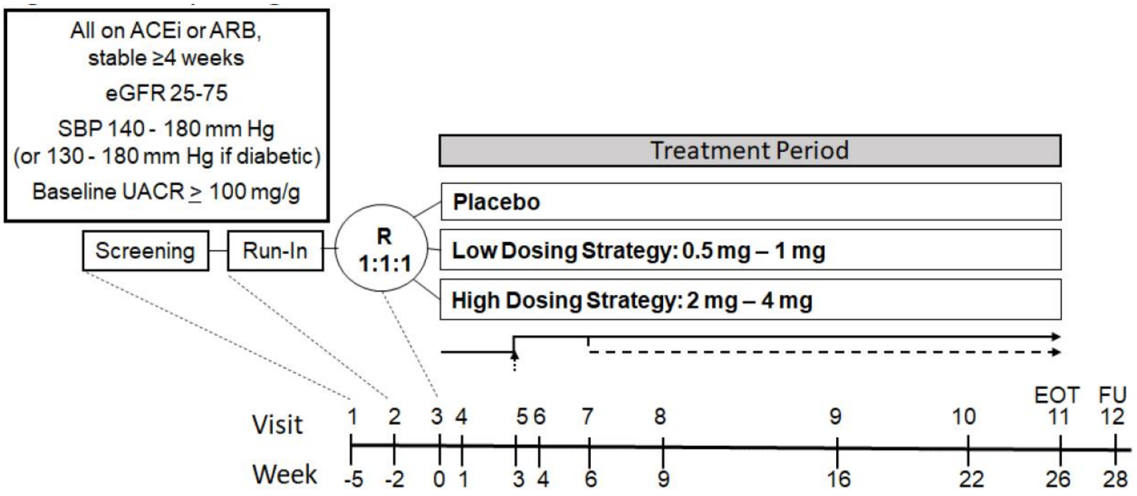

Visit/week	1/-5	2/-2	3/0	4/1	5/3	6/4	7/6	8/9	9/16	10/22	11/26	12/28
BP	Δ	Δ	Δ	Δ	Δ	Δ	Δ	Δ	Δ	Δ	Δ	Δ
UACR		Δ							Δ		Δ	Δ
Safety	Δ	Δ	Δ	Δ	Δ	Δ	Δ	Δ	Δ	Δ	Δ	Δ
PK									Δ		Δ	
PD			Δ						Δ		Δ	Δ

3.1.1. Treatment Assignments

At Visit 3 (Randomization Visit), patients who meet all inclusion/exclusion criteria will be randomly assigned using an automated IRT system in a 1:1:1 ratio to 1 of the 3 treatment groups receiving the following study drug:

- Placebo group: to take placebo for CIN-107
- Low dosing strategy group: to take CIN-107 0.5 mg and may up-titrate to take CIN-107 1 mg at Week 3 (Visit 5)
- High dosing strategy group: to take CIN-107 2 mg and may up-titrate to take CIN-107 4 mg at Week 3 (Visit 5)

It is anticipated that approximately 174 patients will be randomized. Randomization information will be concealed from the Investigators, the patients, and the study team until the end of the study, with the exception of an emergency situation involving a patient that requires unblinding of the treatment assignment (see Section 5.4 in protocol). Randomization will be stratified by Sodium-glucose cotransporter 2 (SGLT2) inhibitor use, baseline systolic blood pressure (SBP) (≤ 155 mmHg or > 155 mmHg) and CKD category (eGFR ≤ 45 mL/min/1.73 m2 or > 45 mL/min/1.73 m^2).

3.1.2. Planned Analyses

This is a Randomized, Double-Blind, Placebo-Controlled, Multicenter, Parallel-Group study. Hence separate blinded and unblinded teams will be involved at AZ and PXL until formal database lock at the end of the study. The blinded team will remain blinded until database lock. The blinding and unblinding plans are detailed out in a separate document (see Blinding Maintenance Plan).

3.2. Statistical Hypothesis

3.2.1. Primary Efficacy Analysis

The primary efficacy analysis will compare the mean change in seated (SBP) from baseline to Week 26 of pooled CIN-107 and placebo using the mITT population. A MMRM will be used to perform this analysis, and an estimate of the treatment difference in the mean change at Week 26 will be generated.

The null hypothesis is defined as:

H0: The true means of the treatment difference at Week 26 in pooled CIN-107 group and placebo group are equal at 2-sided 0.05 significance level.

See Section 4.6.2 for the detailed explanation of covariates, fixed effects and covariance structure for MMRM model.

3.2.2. Secondary Efficacy Analysis

The secondary efficacy analysis will compare the mean change in seated SBP from baseline to Week 26 of each assigned (high-dose or low-dose) CIN-107 dosing group and placebo using the modified mITT population. A MMRM will be used to perform this analysis, and an estimate of the treatment difference in the mean change at Week 26 will be generated.

The null hypotheses for CIN107 high-dosing strategy group vs placebo is defined as:

H0: The treatment difference at Week 26 in assigned high-dosing CIN-107 strategy group and placebo group are equal at 2-sided 0.05 significance level.

The null hypotheses for CIN107 low-dosing strategy group vs placebo is defined as:

H0: The treatment difference at Week 26 in assigned low-dosing CIN-107 strategy group and placebo group are equal at 2-sided 0.05 significance level.

To protect the overall alpha level, the secondary efficacy endpoints will be tested in a hierarchical manner, each at a 2-sided 0.05 level of significance. The change from baseline of SBP in pooled CIN-107 dosing strategy group compared to placebo at Week 26 will be checked at the 0.05 significance level first. If it is significant, continue with checking secondary endpoints in the order described below (see Figure 2), otherwise stop checking.

Figure 2: The Hierarchical Test Procedure

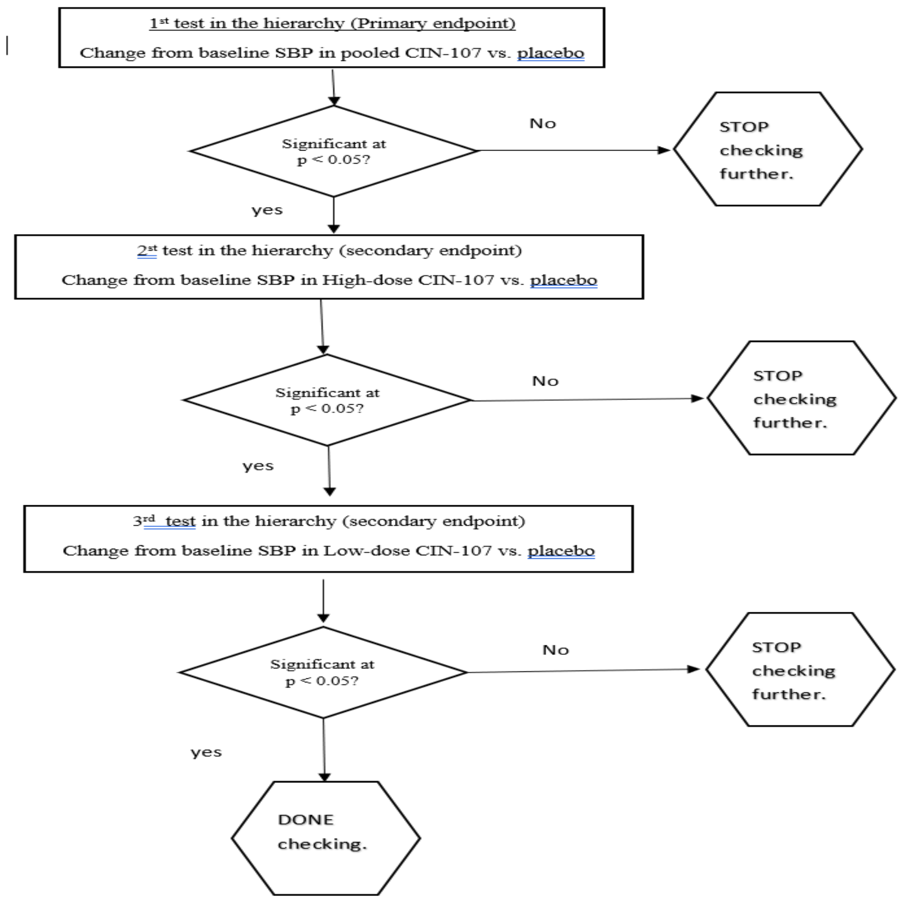

The primary and secondary efficacy endpoints will be analyzed for the following subgroups:

Baseline eGFR: < 45, ≥ 45

< 30, 30 - < 45, 45 -< 60, ≥ 60

SBP: ≤ 155 mmHg, > 155 mmHg

< 140 mmHg, 140 - < 160 mmHg, ≥ 160 mmHg

Type 2 diabetes at baseline: Yes or No

Diuretics used at baseline: Yes or No

SGLT2 inhibitor used at baseline: Yes or No

Baseline Potassium: ≤ 4.5, > 4.5

≤ 4.0, > 4.0 - 4.5, > 4.5 - 4.8, > 4.8

Glucagon-Like Peptide-1 Receptor Agonists used at baseline: Yes or No

Plasma Renin Activity at baseline: < 1, ≥ 1

BMI at baseline: < 30, ≥ 30

Serum aldosterone at baseline: < 6, ≥ 6

Number of anti-hypertensive drugs at baseline: 1 or 2, 3 or more

3.2.3. Exploratory Analysis

Exploratory efficacy analyses will compare:

- The percentage of patients achieving SBP < 130 mmHg at Week 26 in patients assigned to the high-dose strategy group.
- The percentage of patients achieving SBP < 130 mmHg at Week 26 in patients assigned to the low-dose strategy group.
- The change from baseline in DBP measured by AOBPM with each dosing strategy of CIN-107 compared to placebo at Week 26.
- The percentage of change from baseline in UACR with each dosing strategy of CIN-107 compared to placebo at Week 26.

An MMRM model will be used to perform the analyses for change from baseline in DBP measured by AOBPM and percentage change from baseline in UACR (see Section 4.8.3 for details of the analysis model). The percentage change from baseline in UACR will be analyzed

for the following subgroups, by treatment groups (CIN-107 pooled strategy group, CIN-107 high doing group and CIN-107 low dosing group) using MMRM model.

Baseline eGFR: < 45, ≥ 45

SBP: ≤ 155 mmHg, > 155 mmHg

Type 2 diabetes at baseline: Yes or No

SGLT2 inhibitor used at baseline: Yes or No

Baseline Potassium: ≤ 4.5, >4.5

BMI at baseline: < 30, ≥ 30

Serum aldosterone at baseline: < 6 , ≥ 6

Plasma Renin Activity at baseline: < 1, ≥ 1

Achieving SBP < 130 mmHg after 26 weeks of treatment will be analyzed using logistic regression model.

The following additional exploratory efficacy analyses will be explored using MMRM model (see Section 0 for details).

Change from baseline in eGFR at Week 26 in patients assigned to the high dose strategy group.

Change from baseline in eGFR at Week 26 of treatment in patients assigned to the low dose strategy group.

3.2.4. PK Analysis

Individual plasma concentration data for CIN-107 will be listed and summarized by CIN-107 treatment groups, and by PK sample collection timepoint for the PK Population.

3.2.5. PD Analysis

The following PD variables will be listed and summarized for the PD Population:

- Serum and/or plasma parameters: aldosterone concentration, 11-deoxycorticosterone, corticosterone, 11-deoxycorticosterone, 18-OH-corticosterone, cortisol, 11-deoxycortisol, NT-proBNP, PRA, direct renin concentration, angiotensinogen, angiotensin II, Serum PICP, the ratio of PICP to collagen type-1 C-terminal telopeptide.

- 24-hour urine parameters: aldosterone, albumin, creatinine, renin, KIM-1, cystatin C, NGAL, protein.

The MMRM model will be used to analyze the changes from baseline over time with each dose strength of CIN-107 (CIN-107 pooled strategy group, CIN-107 high dosing group and CIN-107 low dosing group) compared to placebo at Week 26 in selected PD variables listed below:

Serum and/or plasma parameters:

- aldosterone concentration
- 11-deoxycorticosterone
- Cortisol
- 11--deoxycortisol
- NT-proBNP
- Direct renin concentration

24-hour urine parameters:

- Albumin
- Creatinine

No adjustment for multiple comparisons will be made. Nominal p-values will be used to examine any trends in these endpoints.

The following relationship objectives will be explored using plots:

- The relationship between percentage of change in UACR and changes in aldosterone and renin levels with each dose group of CIN-107 compared with placebo at Week 26.
- The relationship between change in UACR and changes in BP with each dose group of CIN-107 compared with placebo at Week 26.
- The relationship between the change in eGFR and changes in BP with each dose group of CIN-107 compared with placebo at Week 26.

The PK/PD objectives not part of the CSR will be explained outside the SAP.

4. STATISTICAL METHODS

4.1. Data Quality Assurance

All tables, figures, and data listings will be independently checked for consistency, integrity, and in accordance with standard Parexel procedures.

4.2. General Presentation Considerations

Demographics, baseline characteristics, efficacy and PD endpoints will be summarized by assigned (randomized) treatment groups for the respective study populations. Safety and PK endpoints will summarized for safety population and PK population respectively, by actual treatment groups that is same as randomized treatment except in cases when the patient took only the wrong treatment for the duration of the study. In that case, patient will be summarized under the actual treatment the patient received.

Continuous data will be summarized using descriptive statistics (the number of patients [n], mean, SD, median, 25th and 75th percentiles [where appropriate], minimum and maximum unless otherwise stated). For log-transformed data it is more appropriate and will present gmean, geometric CV, median, minimum and maximum.

For continuous data, the minimum and maximum will be reported to the same number of decimal places as the raw data recorded in the database. The mean, median, lower quartile and upper quartile will be reported to 1 more decimal place than the raw data recorded in the database. The SD and geometric CV will be rounded to 2 additional decimal places compared to the original data. Minimum and maximum will be displayed with the same accuracy as the original data. The maximum number of decimal places reported will be 4 for any summary statistic.

Categorical data will be summarized in terms of the number of patients providing data at the relevant time point (n), frequency counts and percentages. Percentages will not be presented for 0 (zero) counts. Unless otherwise stated, percentages will be calculated using the number of patients included in the analysis set for that treatment group as denominator and presented to 1 decimal place.

CIs and p-values, when presented, will generally be constructed at the 2-sided alpha level unless otherwise specified. P-values greater than or equal to 0.001, will be presented to 3 decimal places. P-values less than 0.001 will be presented as “< 0.001”. and p-values greater than 0.999 will be presented as “> 0.999.” CIs will be presented to one more decimal place than the raw data.

Date variables are formatted as YYYY-MM-DD for presentation. Time is formatted in military time as HH:MM for presentation.

For assessments on the day of first dose where time is not captured, a nominal pre-dose indicator, if available, will serve as evidence that the assessment occurred prior to first dose. Assessments on the day of first dose where neither time nor a nominal pre-dose indicator are captured will be considered prior to first dose if such procedures are required by the protocol to be conducted before first dose.

For efficacy, safety endpoints, baseline will be the last non missing value obtained prior to the first dose/administration of study medication, unless otherwise specified (see section 4.3.1 for details) and any information taken after first dose/administration of study medication will be regarded as post baseline information. When time is not available, any observation on the same day as first dose will be assumed to be before first dose and may qualify as the baseline value. Where efficacy and safety data are summarized over time, study day will be calculated in relation to date of first treatment. If no value exists before the first dose/administration, then the baseline value will be treated as missing. Unless time variables indicate otherwise, AEs occurring on the day of first dose will be assumed to have occurred after first dose and will be assumed to be TE.

In all summaries:

Change from baseline = post-baseline value - baseline value.

$$\text{Percent change from baseline} = \frac{\text{post-baseline value - baseline value}}{\text{baseline value}} \times 100.$$

For any variable patient to log transformation, the back transformed change from baseline, calculated and summarized on the log scale, will be presented as the 'GMR'. Percentage change will then be calculated as (GMR – 1) x 100.

Wherever possible, data will be decimal aligned.

Treatment differences will be calculated as (Active - Placebo), so that positive values indicate increases relative to placebo and negative values indicate decreases relative to placebo.

4.3. General variables

4.3.1. Baseline

Measurements of efficacy and safety variables recorded prior to the first dose of double-blind study drug administration will constitute pre-dose measurements.

For blood pressure measurements (SBP, DBP), baseline is defined as the average of the 3 measurements taken prior to randomization at Visit 3. If only 2 of the triplicate measurements are

available, then mean of those 2 non-missing measurements will be used as baseline. If only 1 non-missing value is available, that value will be considered as baseline. If any of the triplicate measurements is repeated due to technical issues at the same visit, the repeat measurement will be used for calculating the average.

For UACR measurements, baseline is defined as geometric mean of the 3 samples returned at Visit 2. In case triplicate measurements are not available, the geometric mean of the available measurements will be taken as baseline. For all UACR measurements (baseline and post-baseline), the latest recorded date/time of the UACR samples collected will be considered as the assessment date and it will be used for visit windowing.

For all other laboratory variables, vital signs, and PD variables, the baseline will be the latest recorded measurement prior to the first dose of the study drug. If more than 1 measurement is taken on the same day for a particular laboratory or vital signs parameter, the value with the later time (if available) will be used. Otherwise, the average of the measurements taken on the same day will be used as the baseline.

4.3.2. Relative Day

In this study, the study drug is defined as CIN-107 or placebo.

The relative day for an assessment will be calculated from the first dosing date of the study drug and derived as follows:

- For days prior to the first dose: Assessment Date – first dosing date
- For post-dose relative days: Assessment Date – first dosing date +1

The relative day will be included in relevant listings.

4.3.3. Visit Windows

The visit windows are provided in Table 2. Visit windowing will be applied for both efficacy and safety analyses. If multiple visits occur in a time window, the one closest to the target window will be used for analyses. If the two visits have the same distance from a target window, the later one will be used. If two or more visits have the same date and time (or a date but no time) then the average of these visits will be used. Both unscheduled and scheduled visit values will be considered (with no preference) for use in the analyses using the rules above.

Table 2: Visit Windows for Efficacy, Safety, PD, and PK Endpoints

Study Period	Analysis Visit	Target Day	Assessment Window Study Day
Screening	Week -4	Day -28	-28 to -22
	Week -2	Day -14	-21 to -7
Double-Blind Treatment	Week 0	Day 1	Latest value prior to dosing [e]
	Week 1	Day 7	2 to 14
	Week 3	Day 21	15 to 24
	Week 4	Day 28	25 to 35
	Week 6	Day 42	36 to 52
	Week 9	Day 63	53 to 87
	Week 16	Day 112	88 to 133
	Week 22	Day 154	134 to 168
EOT	Week 26	Day 182	169 to 189
FU	Week 28	Day 196	Day 190 onwards

[e] For UACR, baseline is GM of the triplicate samples returned on Week -2 (Visit 2).
For SBP and BP, is the average of the triplicate measurements taken on Week 0 (Visit 3) prior to randomization.
For other safety parameters, baseline is the latest value prior to first dosing of the treatment.

For all efficacy endpoints, the measurements taken up to Week 26 (± 7 days) will be included.

4.3.4. Handling Missing Data

In general, other than the below described, or where otherwise specified in the particular analysis, missing data will not be imputed and will be treated as missing.

All efficacy and safety analyses will be performed using available data following the methods for ICEs described in Section 2.2.1, and no imputation will be performed on missing data with the exception of the binary endpoint of patients reaching < 130 mmHg. For this endpoint, patients who have missing data at the endpoint (Week 26) will be assumed to be non-responders.

4.3.4.1. Missing or Partial Dates

All listings will report dates as reported on the eCRF and will not include imputed dates.

4.3.4.2. Imputation rules for Prior or Concomitant Medication Dates

Missing Medication Start or End Date

If the medication start date is missing, and the medication stop date is on or after the first dose of study medication, then the medication start date will be imputed as the date of the first dose of study medication.

If the medication start date is missing, and the medication stop date is not missing and before the first dose of study medication, then the medication start date will be imputed as the medication stop date.

Partial Medication Start Date

Missing day and month

- If the year of the incomplete start date is the same as the year of the date of the first dose of study medication, then the day and month of the date of the first dose of study medication will be assigned to the missing fields.
- If the year of the incomplete start date is before the year of the date of the first dose of study medication, then December 31 will be assigned to the missing fields.
- If the year of the incomplete start date is after the year of the date of the first dose of study medication, then January 1 will be assigned to the missing fields.

Missing month only

- The day will be treated as missing and both month and day will be replaced according to the above procedure.

Missing day only

- If the month and year of the incomplete start date are the same as the month and year of the date of the first dose of study medication, then the day of the first dose of study medication will be assigned to the missing day.
- If either the year is before the year of the date of the first dose of study medication or if both years are the same but the month is before the month of the date of the first dose of study medication, then the last day of the month will be assigned to the missing day.
- If either the year is after the year of the date of the first dose of study medication or if both years are the same but the month is after the month of the date of the first dose of study medication, then the first day of the month will be assigned to the missing day.

Partial Medication Stop Date

If a medication stop date is missing and the ongoing status is also missing, then the medication is assumed to be ongoing.

If the imputed medication stop date is before the study medication start date (whether imputed or non-imputed), then the imputed medication stop date will be equal to the study medication start date.

Missing day and month

- If the year of the incomplete stop date is the same as the year of the date of the last dose of study medication, then the day and month of the date of the last dose of study medication will be assigned to the missing fields.
- If the year of the incomplete stop date is before the year of the date of the last dose of study medication, then December 31 will be assigned to the missing fields.
- If the year of the incomplete stop date is after the year of the date of the last dose of study medication, then January 1 will be assigned to the missing fields.

Missing month only

- The day will be treated as missing and both month and day will be replaced according to the above procedure.

Missing day only

- If the month and year of the incomplete medication stop date are the same as the month and year of the date of the last dose of study medication, then the day of the last dose of study medication will be assigned to the missing day.
- If either the year is before the year of the date of the last dose of study medication or if both years are the same but the month is before the month of the date of the last dose of study medication, then the last day of the month will be assigned to the missing day.

If either the year is after the year of the date of the last dose of study medication or if both years are the same, but the month is after the month of the date of the last dose of study medication, then the first day of the month will be assigned to the missing day.

4.3.4.3. Imputation Rules for AE Dates

Missing AE Start Date

If the AE start date is missing, and the AE stop date is on or after the first dose of study medication, then the AE start date will be imputed as the date of the first dose of study medication.

If the AE start date is missing, and the AE stop date is not missing and before the first dose of study medication, then the AE start date will be imputed as the stop date.

Partial AE Start Date

Missing day and month

- If the year is the same as the year of the date of the first dose of study medication, then the day and month of the date of the first dose of study medication will be assigned to the missing fields.
- If the year is before the year of the date of the first dose of study medication, then December 31 will be assigned to the missing fields.
- If the year is after the year of the date of the first dose of study medication, then January 1 will be assigned to the missing fields.

Missing month only

- The day will be treated as missing and both month and day will be replaced according to the above procedure.

Missing day only

- If the month and year are the same as the month and year of the date of the first dose of study medication, then the day of the first dose of study medication will be assigned to the missing day.
- If either the year is before the year of the date of the first dose of study medication or if both years are the same but the month is before the month of the date of the first dose of study medication, then the last day of the month will be assigned to the missing day.
- If either the year is after the year of the date of the first dose of study medication or if both years are the same but the month is after the month of the date of the first dose of study medication, then the first day of the month will be assigned to the missing day.
- If the imputed AE start date is after the AE stop date, then the imputed AE start date will be population to the AE stop date.

4.4. Software

All report outputs will be produced using SAS® Version 9.4 or later in a secure and validated environment.

4.5. Study Patients

4.5.1. Disposition of Patients

A summary of patient disposition will be provided for all patients who signed informed consent, by treatment group and overall.

The number and percentage of the patients in the following categories will be summarized:

- All patients screened.
- Screen failed.
- Reasons for screen failures.
- The patients who were randomized.
- Completed study treatment.
- Did not complete study treatment with reasons for premature discontinuation of study drug.
- Completed study.
- Did not complete the study with reasons for premature discontinuation of study.

Only the pooled number will be provided for all patient screened, screen failures, and reasons for screen failure. For the status of study drug and study completion and reasons for premature discontinuation, the number and percentage of patients in each category will be provided. The denominator for the percentage calculation will be the total number of patients in the ITT population in each treatment group.

The reasons for premature study discontinuation will be listed by PID number for each treatment group.

The reasons for screen failure will also be provided by PID number in ascending order.

4.5.2. Protocol Deviations

A protocol deviation is any noncompliance with the clinical trial protocol, ICH GCP, or SOP requirements. The noncompliance may be either on the part of the patient, the Investigator, or the study site staff. Protocol deviations occurring after patients entered the study are documented during the routine monitoring. IPDs are defined as those deviations from the protocol likely to have heavy impact on the interpretation of any analysis based on addressing the primary and secondary objectives of the trial or those may significantly affect a patient's rights, safety, or wellbeing.

The general protocol deviation categories will be programmatically derived from the eCRF data. These deviations will be reviewed and assessed on a case-by-case basis by AZ to determine importance. Deviations considered to be important will be listed and discussed in the CSR as appropriate. All decisions on importance will be made ahead of database lock for the primary analysis and will be documented prior to the primary analysis being conducted. Additional non-programmable protocol deviations identified during site visits will be listed as an appendix in the CSR. A summary of the number and percentage of patients with an IPD by deviation category will be provided based on the ITT population. Also, a by-patient listing of IPDs will be provided.

4.5.3. Analysis Sets

The patients who are included in and excluded from each analysis set (see Table 3) based on ITT population will be summarized, with reasons for exclusions from each analysis set. The patients excluded from analysis sets will also be listed.

Table 3: Analysis Sets

Analysis set	Definition
ITT population	All patients randomized into the study. Treatment classification will be based on the randomized treatment assignment.
mITT population	The mITT population will include all patients in the ITT population who receive at least 1 dose of any study drug. Treatment classification will be based on the randomized treatment assignment.
Safety population	All patients who receive at least one dose of any study drug. Treatment classification will be based on the actual treatment received. The safety population will be the primary population used for the safety analyses.
PK population	All patients in the ITT population who received at least 1 dose of any study drug and haveat least one quantifiable plasma concentration.

PD population	All patients in the ITT population who received at least 1 dose of any study drug and have at least one quantifiable PD parameter value.

4.5.4. Demographics and Baseline Characteristics

Demographic and baseline characteristics will be summarized for ITT population by assigned treatment groups, pooled CIN-107 treatment groups, placebo and overall using descriptive statistics for continuous variables, number and percentage of patients for categorical variables.

Demographic characteristics summarized and listed will include age (categorical [18 – 65, 66 – 75 and > 75 years] and continuous, sex, race, ethnicity, body weight (kg), height (cm), BMI at baseline (categorical [< 30 kg/m^2, ≥ 30 kg/m^2] and continuous) and childbearing potential.

The following baseline characteristics summarized and listed: SBP (categorical [≤ 155 mmHg, > 155 mmHg] and continuous), DBP, eGFR (categorical [< 30, 30 to 44, 45 to 59, ≥ 60 mL/min/1.73m^2] and continuous), serum aldosterone (categorical [≥ 6 or < 6 ng/dL] and continuous), PRA (categorical [< 1 ng/mL/hr, ≥ 1 ng/mL/hr] and continuous), ARR, UACR (categorical [normal, microalbuminuria, and macroalbuminuria] and continuous), Type 2 diabetes status (Yes or No), heart failure (Yes or No), use of ACEi/ARB (ACEi only, ARB only, and Both) use of SGLT2 inhibitor (Yes or No), and background antihypertensive medication use at baseline (Yes or No), number of background antihypertensive medications use at baseline (1, 2, 3, and > 3). Background hypertensive medications will be summarized for medications other than ACEi and ARBs.

4.5.5. Medical History

Medical history collected at screening will be coded using MedDRA Version 24.1.

Medical history will be summarized by SOC, preferred term for each assigned treatment group and overall, for ITT population. Patients who report 2 or more medical history items that are coded to the same SOC and preferred term will be counted only once by the unique coded term in the summary. The summary will be provided for the ITT population.

A listing of medical history will be provided.

4.5.6. Surgical History

Surgical history collected at screening will be coded using MedDRA Version 24.1.

Surgical history will be summarized by SOC, preferred term for each assigned treatment group and overall, for ITT population. Patients who report 2 or more medical history items that are

coded to the same SOC and preferred term will be counted only once by the unique coded term in the summary. The summary will be provided for the ITT population.

A listing of surgical history will be provided.

4.5.7. Prior and Concomitant Medication

Information on any medications that the patient is receiving at the time of enrolment and all concomitant medications given up to end of study, with reasons for the treatment, dates of administration and the dosage will be recorded in the eCRF.

For the purpose of inclusion in prior and/or concomitant medication summaries, incomplete medication start and stop dates will be imputed as detailed in Section 4.3.4

- Prior medications are those taken prior to study treatment with a stop date prior to first dose of study treatment.
- Concomitant medications are those with a stop date on or after the first dose date of study treatment (and could have started prior to or during treatment).
- Post treatment medications are those with a start date after the last dose date of study treatment.

Prior and concomitant medications will both be summarized for ITT population by ATC drug class Level 3 and preferred name using the number and percentage of patients for each assigned treatment group and overall based on the ITT population. ATC level 4 will be used to summarize the data. ATC level 3 will be used if ATC level 4 code missing or otherwise unavailable for a particular medication. A patient reporting the same medication more than once will be counted only once within each preferred name. The summary will be ordered alphabetically by ATC medical class and then by preferred drug name in order of descending overall frequency within each ATC drug class. For drugs with the same frequency, sorting will be done alphabetically.

All prior and concomitant medications will be listed by PID and sorted by administration date in chronological order for each treatment group.

4.6. Primary Efficacy Analysis

4.6.1. Definition of Endpoint(s)

The primary efficacy endpoint is the change in mean seated SBP from baseline to Week 26 of pooled CIN-107 and placebo using the mITT population. The MMRM will be used to perform the analysis.

All available data will be used, including data collected after receipt of other treatment for hypertension (see Table 1 describing estimands and ICEs), and all missing data are assumed to be missing at random.

Summary statistics (sample size, mean, SD, median, Q1, Q3, minimum and maximum) of the seated SBP value, change from baseline percentage change from baseline will be provided for the individual assigned treatment groups, pooled CIN-107 and placebo groups by each visit until Week 28 (Visit 12).

4.6.2. Main Analytical Approach

An MMRM will be used. Change from baseline in mean seated SBP at all planned post-baseline visits up to Week 26 (Visit 11) will be included. The analysis will include fixed effects for treatments (pooled CIN-107 treatment strategies and placebo), visit, stratification variables (SGLT2 inhibitor use and CKD category), and the treatment-by-visit interaction, along with a covariate of the baseline seated SBP value and the baseline seated SBP by visit interaction. The stratification variable of seated baseline SBP (≤ 155 mmHg or > 155 mmHg) will not be included in the model. The treatment variable in the model will include 2 levels: the combined low dose and high dose strategy groups of CIN-107 along with placebo.

The REML estimation will be used with an UN covariance matrix. If model convergence issues arise, the following structures will be used in the following order until convergence is achieved: Toeplitz, then AR(1) and finally CS. The least square means, standard errors for each treatment (pooled CIN-107 and placebo) and the estimated treatment differences for the treatment comparisons (pooled CIN-107 vs placebo group) will be presented together with 95% CIs, along with p-values for the treatment comparisons for all scheduled visits for seated SBP until Week 26. The effect at Week 26 will be tested with 0.05 2-sided alpha. All other p-values will be nominal.

A line plot of the least square means and estimated treatment differences for the treatment comparisons with corresponding 95% CIs will also be produced.

For this and all MMRM models, only data corresponding to the selected value within the windows for scheduled visits for the analyzed variable will be included in the model, even if data exists for visits where the variable was not to be collected. The follow up visit window data will not be included in the models.

4.7. Secondary Efficacy Analysis

4.7.1. Definition of Endpoint(s)

The following secondary efficacy endpoints will evaluated in the hierarchical order only if the primary efficacy endpoint is statistically significant at 2-sided 0.05 significant level. Low-dose strategy group vs placebo will be checked only if high-dose strategy group vs placebo is significant.

- The change from baseline of SBP on CIN-107 compared with placebo at Week 26 in patients assigned to the high-dose strategy group
- The change from baseline of SBP on CIN-107 compared with placebo at Week 26 in patients assigned to the low-dose strategy group

4.7.2. Main Analytical Approach

An MMRM will be used. Change from baseline in mean seated SBP at all planned post-baseline visits up to Week 26 (Visit 11) will be included. The analysis will include fixed effects for treatments (CIN-107 treatment strategies and placebo), visit, stratification variables (SGLT2 inhibitor use and CKD category), and the treatment-by-visit interaction, along with a covariate of the baseline seated SBP value and the baseline seated SBP by visit interaction.

The REML estimation will be used with an UN covariance matrix. If model convergence issues arise, the following structures will be used in the following order until convergence is achieved: Toeplitz, then AR(1) and finally, CS. The least square means, standard errors for each treatment (CIN-107 and placebo) and the estimated treatment differences for the treatment comparisons (CIN-107 vs placebo group) will be presented together with 95% CIs, along with p-values for the treatment comparisons for all scheduled visits for DBP until Week 26. The effect at Week 26 will be tested with 0.05 2-sided alpha. All other p-values will be nominal.

A line plot of the least square means and estimated treatment differences for the treatment comparisons with corresponding 95% CIs will also be produced.

4.8. Exploratory Endpoints

All available data will be used for exploratory endpoints, including data collected after receipt of other treatment for hypertension (see Table 1 describing estimands and ICEs), and all missing data are assumed to be missing at random.

Summary statistics (sample size, mean, SD, median, Q1, Q3, minimum and maximum) of the respective exploratory endpoints (unless specified otherwise) at each scheduled assessment, and

change from baseline will be provided for individual assigned treatment groups, pooled CIN-107 and placebo groups until Week 28 (Visit 12).

For all exploratory efficacy analysis using MMRM model, the REML estimation will be used with an UN covariance matrix. If model convergence issues arise, the following structures will be used in the following order until convergence is achieved: Toeplitz, then AR(1) and finally, CS. The least square means, standard errors for each treatment (CIN-107 and placebo) and the estimated treatment differences for the treatment comparisons (CIN-107 vs placebo group) will be presented together with 95% CIs, along with p-values for the treatment comparisons for all scheduled visits until Week 26. The effect at Week 26 will be tested with 0.05 2-sided alpha.

All other p-values will be nominal.

4.8.1. Percentage of Patients Achieving SBP < 130 mmHg at Week 36 in CIN-107 Treatment Groups

Patients achieving SBP < 130 mmHg after 26 weeks of treatment compared with placebo will be analyzed using logistic regression models controlling for treatment (CIN-107 treatment strategies and placebo), baseline SBP value and stratification variables (SGLT2 inhibitor use and CKD category) for the following endpoints; odds ratio of patients achieving along with 2-sided 95% CI will also be summarized. Two separate models will be fit. One for pooled CIN-107 treatment groups vs. placebo and one for low and high dosing strategy groups vs. placebo. Patients with missing values at week 26 will be imputed as non-responders.

- The percentage of patients achieving SBP < 130 mmHg at Week 26 in patients assigned to CIN-107 high-dosing strategy group
- The percentage of patients achieving SBP < 130 mmHg at Week 26 in patients assigned to CIN-107 low-dosing strategy group
- The percentage of patients achieving SBP < 130 mmHg at Week 26 in patients assigned to CIN-107 pooled treatment group

A forest plot for the odds ratios with 95% CI for patients achieving SBP < 130 mmHg by Week 26 for each dosing strategy (including CIN-107 pooled) compared with placebo will be plotted.

4.8.2. Change from baseline in DBP measured by AOBPM with each dosing strategy of CIN-107 compared to placebo at Week 26.

An MMRM will be used to analyze change from baseline in DBP at all planned post-baseline visits up to Week 26 (Visit 11). The analysis will include fixed effects for treatments (CIN-107

treatment strategies and placebo), visit, stratification variables (SGLT2 inhibitor use and CKD category), and the treatment-by-visit interaction, along with a covariate of the baseline DBP value and the baseline DBP by visit interaction. The least square means, standard errors for each treatment (CIN-107 and placebo) and the estimated treatment differences for the treatment comparisons (CIN-107 vs placebo group) will be summarized together with 95% CIs, along with p-values for the treatment comparisons for all scheduled visits until Week 26. The effect at Week 26 will be tested with 0.05 2-sided alpha.

All other p-values will be nominal.

4.8.3. The Percentage of Change from Baseline in UACR with Each Dosing Strategy of CIN-107 Compared to Placebo at Week 26.

Summary statistics of UACR will also be presented by baseline SGLT2 inhibitor use (Yes or No) and baseline eGFR category (≤ 45mL/mim/1.73m^2, > 45 mL/min/1.73 m^2).

An MMRM will be used to analyze log transformed UACR overall and log transformed UACR by subgroups specified in Section 3.2.3 at all planned post-baseline visits up to Week 26 (Visit 11). The analysis will include fixed effects for treatments (CIN-107 treatment strategies and placebo), visit, stratification variables (SGLT2 inhibitor use, baseline SBP category and CKD category), and the treatment-by-visit interaction, along with a covariate of the log transformed baseline UACR value and the log (baseline UACR) by visit interaction.

Percentage changes from baseline for each treatment (CIN-107 and placebo) and the estimated percentage change in the GMR ratios for the treatment comparisons (CIN-107 vs placebo group) will be summarized together with 95% CIs, along with p-values for Week 16 and Week 26 (Visit 11).

4.8.4. Change from Baseline in eGFR at Week 26

Summary statistics of eGFR will be presented by baseline SGLT2 inhibitor use (Yes or No) and baseline eGFR category (≤ 45mL/min/1.73m^2, > 45 mL/min/1.73 m^2).

An MMRM will be used to analyze change from baseline in eGFR at all planned post-baseline scheduled assessments up to Week 26 (Visit 11). The analysis will include fixed effects for treatments (CIN-107 treatment strategies and placebo), visit, stratification variables (SGLT2 inhibitor use, baseline SBP category), and the treatment-by-visit interaction, along with a covariate of the baseline eGFR value and baseline eGFR by visit interaction.

The least square means, standard errors for each treatment (CIN-107 and placebo) and the estimated treatment differences for the treatment comparisons (CIN-107 vs placebo group) will

be summarized together with 95% CIs, along with p-values for the treatment comparisons for all scheduled visits until Week 26. The effect at Week 26 will be tested with 0.05 2-sided alpha.

All other p-values will be nominal.

4.8.5. To Determine the Changes from Baseline in the PD Variables of Each Dose Strength of CIN-107 Compared to Placebo at Week 26

All PD variables listed in Section 3.2.5 will summarized and listed.

An MMRM model will be used to analyze the changes from baseline over time with each dose strength of CIN-107 (CIN-107 pooled strategy group, CIN-107 high dosing group and CIN-107 low dosing group) compared to placebo at Week 26 in selected PD variables listed below:

Serum and/or plasma parameters:

- Aldosterone concentration
- 11-deoxycorticosterone
- Cortisol
- 11--deoxycortisol
- NT-proBNP
- Direct renin concentration

24-hour urine parameters:

- Albumin
- Creatinine

Each PD parameter value will be checked for normality before using an MMRM model. The normality of the distribution of the data will be tested using a Shapiro-Wilk test. In case of a two-sided p-value > 0.20, there will be evidence that the data is normally distributed. Additional plots and statistics will be provided to measure the severity of the deviations from normality if necessary. A log transformed parameter value will be used if normality is not satisfied. The results will not be back transformed. The analysis will include fixed effects for treatments (CIN-107 treatment strategies and placebo), visit, stratification variables (SGLT2 inhibitor use, baseline SBP category and CKD category), and the treatment-by-visit interaction, along with a covariate of the baseline PD value or log transformed PD value (depending on normality) and baseline PD value or log (baseline PD value) by visit interaction.

No adjustment for multiple comparisons will be made. Nominal p-values will be used to examine any trends in these endpoints.

The least square means, standard errors for each treatment (CIN-107 and placebo) and the estimated treatment differences for the treatment comparisons (CIN-107 vs placebo group) will be summarized together with 95% CIs, along with p-values for the treatment comparisons for only Week 26 (visit 11). The effect at Week 26 will be tested with 0.05 2-sided alpha.

All other p-values will be nominal.

4.8.6. To Evaluate the Dose-response Relationships of CIN-107 in Patients with uHTN and CKD Using Measures of Safety, PD, and/or Efficacy at Week 26

The following exploratory relationship objectives will be explored by scatter plots for each treatment group:

- The relationship between percentage of change in UACR and changes in aldosterone and with each dose strength of CIN-107 compared with placebo at Week 26.
- The relationship between percentage of change in UACR and changes in renin levels with each dose strength of CIN-107 compared with placebo at Week 26.
- The relationship between percentage change in UACR and changes in SBP with each dose strength of CIN-107 compared with placebo at Week 26.
- The relationship between percentage change in UACR and changes in DBP with each dose strength of CIN-107 compared with placebo at Week 26.
-
- The relationship between the change in eGFR and changes in SBP with each dose strength of CIN-107 compared with placebo at Week 26.
- The relationship between the change in eGFR and changes in DBP with each dose strength of CIN-107 compared with placebo at Week 26.

Each plot will be plotted separately by CIN-107 pooled treatment groups, each of the CIN-107 treatment groups and placebo with a common range for axes for each set of plots to make comparison across treatments easier.

4.9. Sensitivity

The following sensitivity analyses may be explored separately if needed at a later time:

Sensitivity 1: A sensitivity analysis of mean seated SBP change from baseline at Week 26, that uses a hypothetical estimand for ICEs of other anti-hypertensive medications and treatment

discontinuation (ie, exclude data after other anti-hypertensive medication use and after treatment discontinuation) will be performed.

Sensitivity 2: If data permits, a sensitivity analysis of mean seated SBP change from baseline at Week 26 excluding the patients prior to an unblinding event happened in fall of 2022.

Both sensitivity analyses will use an MMRM model including fixed effects for treatments (pooled CIN-107 treatment strategies and placebo), visit, stratification variables (SGLT2 inhibitor use and CKD category), and the treatment-by-visit interaction, along with a covariate of the baseline seated SBP value and the baseline seated SBP by visit interaction.

The stratification variable of seated baseline SBP (≤ 155 mmHg or > 155 mmHg) will not be included in the model. The treatment variable in the model will include 2 levels: the combined low dose and high dose strategy groups of CIN-107 along with placebo.

The REML estimation will be used with an UN covariance matrix. If model convergence issues arise, the following structures will be used in the following order until convergence is achieved: Toeplitz, then AR(1) and finally, CS. The least square means, standard errors for each treatment (pooled CIN-107 and placebo) and the estimated treatment differences for the treatment comparisons (pooled CIN-107 vs placebo group) will be presented together with 95% CIs, along with p-values for the treatment comparisons. The effect at Week 26 will be tested with 0.05 2-sided alpha.

All other p-values will be nominal.

A line plot of the least square means and estimated treatment differences for the treatment comparisons with corresponding 95% CIs will also be produced.

4.10. Safety Analyses

4.10.1. Extent of Exposure

Extent of exposure to CIN-107 will be defined in terms of the number of weeks in the treatment. Exposure (ie, the duration of treatment) to study drug will be assessed as follows:

- Total treatment duration (if study drug discontinued after the last dose > 0 mg) = (min (last dosing date where dose > 0 mg, date of death, date of DCO) – first dosing date +1) /7. The expected total duration of treatment for all patients is 26 weeks.
- The duration of exposure in weeks is categorized into the following groups:

  At least one dose

≥ 3 weeks

≥ 6 weeks

≥ 8 weeks

≥ 16 weeks

≥ 24 weeks

- Patients who are up-titrated at 3 weeks and patients who are subsequently down-titrated.

Summaries of the above variables will be performed on the safety population following the methods described in Section.4.2 Results will be presented by actual treatment group and overall.

4.10.2. Study Drug Compliance

The compliance to study drug will be assessed using the following endpoints:

- Total number of tablets administered. This will be determined by the data collected in the patient supply accountability log in eCRF using the following calculation:
- Total number of tablets administered = sum of tablets dispensed – sum of tablets returned
- The Percent Prescribed tablet compliance. This will be determined by the total number of study drug tablets administered relative to the total amount of study drug tablets specified by protocol for a patient who completes the treatment period in the study. If a patient fails to return a blister pack, it will be assumed that all tablets were taken prior to the end of the last day of study treatment. The prescribed compliance will be expressed as a percentage and calculated using below formula among returned kits:

$$\text{Prescribed Compliance (\%)} = \frac{[\text{ total number of tablets administered } /2]}{[(\text{date of last dose})-(\text{date of first dose})+1]} \text{X } 100$$

- The Percent prescribed compliance categorized into the following categories:

  < 80%

  80% to < 85%

  85% to < 90%

  90% to < 95%

  90% to 100%

  80% to 100%

  100 to 110%

  > 110%

Summaries of the total number of tablets administered and prescribed compliance (both categorical and continuous) from randomization will be presented on the safety population following the methods described in Section 4.2. Results will be presented by, pooled CIN-107 treatment groups, overall and by randomized treatment groups.

Study drug accountability will be listed by patient and by visit.

4.10.3. Adverse Events

AEs and SAEs will be collected from the time of signature of informed consent form and throughout the treatment and follow-up period after treatment discontinuation. AEs be classified by SOC and preferred term according to MedDRA Version 24.1.

AEs will be summarized for all AEs, TEAEs on-study and TEAEs on-treatment.

- All AEs: An AE that newly occurs or worsens in severity from the time informed consent signed.
- TEAE on study: An AE that newly occurs or worsens in severity on or after the first double-blind treatment on Visit 3 (Day 1).
- TEAEs on-treatment: An AE that newly occurs or worsens in severity within the double-blind treatment period of Visit 3 (Day 1) up through and including date of last treatment + 10 days, regardless of, if the patient prematurely discontinued the treatment.

An overview table for all AEs including the number of patients and number of events for the following will be summarized for safety population, by actual treatment and overall:

- All AEs.
- All AEs possibly related to treatment.
- Any AE with outcome of death.
- Any AE with outcome of death, possibly related to treatment.
- Any AE leading to dose interruption.
- Any AE leading to dose interruption, possibly related to treatment.
- Any AE leading to dose discontinuation of treatment.
- Any AE leading to dose discontinuation of treatment, possibly related to treatment.
- Any AE leading to discontinuation of study.
- Any AE leading to discontinuation of study, possibly related to treatment.

- Any SAEs.
- All SAEs, possibly related to treatment.
- Any SAE with outcome of death.
- Any SAE with outcome of death, possibly related to treatment.
- Any SAE leading to dose interruption.
- Any SAE leading to dose interruption, possibly related to treatment.
- Any SAE leading to dose discontinuation of treatment.
- Any SAE leading to dose discontinuation of treatment, possibly related to treatment.
- Any SAE leading to discontinuation of study.
- Any SAE leading to discontinuation of study, possibly related to treatment.
- TE AESI.
- All AEs by maximum severity

If the event relationship to the study medication is missing, the event will be categorized as 'Related'.

Additionally, overall summary of on-study and on-treatment TEAEs will be provided for the safety population.

The following on-study and on-treatment TEAE summaries will be presented separately for the safety population by SOC and preferred term:

- Any AE with outcome of death.
- Any SAEs
- Any TEAEs.
- Any TEAEs possibly related to treatment.
- Any TEAE with outcome of death.
- Any TEAE with outcome of death, possibly related to treatment.
- Non-serious TEAEs occurring on-study in more than 5% of patients.
- Any TEAE leading to dose interruption.
- Any TEAE leading to dose interruption, possibly related to treatment.
- Any TEAE leading to dose discontinuation of treatment.
- Any TEAE leading to dose discontinuation of treatment, possibly related to treatment.
- Any TEAE leading to discontinuation of study.

- Any TEAE leading to discontinuation of study, possibly related to treatment.
- Any TESAE.
- Any TESAE with outcome of death.
- Any TESAE with outcome of death , possibly related to treatment.
- Any TESAE leading to dose interruption.
- Any TESAE leading to dose interruption, possibly related to treatment.
- Any TESAE leading to dose discontinuation of treatment.
- Any TESAE leading to dose discontinuation of treatment, possibly related to treatment.
- Any TESAE leading to discontinuation of study.
- Any TESAE leading to discontinuation of study, possibly related to treatment.
- TE AESI.
- . All TEAEs by maximum severity.

Additionally, a table listing will be provided for the following:

- That includes all AEs (including pre-treatment and any time after treatment) that lead to death.
- All AEs leading to discontinuation of treatment.
- All SAEs.
- All SAEs with outcome of death.
- All TEAEs on-study leading to discontinuation of treatment.
- All TEAEs on-treatment leading to discontinuation of treatment.
- All TESAEs on-study.
- All TESAEs on-study with outcome of death.
- All TESAEs on-study leading to discontinuation of treatment.
- All TESAEs on-treatment.
- All TESAEs on-treatment with outcome of death.
- All TESAEs on-treatment leading to discontinuation of treatment.

Treatment emergent AESIs include the following:

- Hypotension events that require clinical intervention.

- Abnormal potassium laboratory values that require clinical invention.
- Abnormal sodium laboratory values that require clinical intervention.

Additionally:

- All AEs will be summarized by decreasing frequency of events by preferred term.
- The preferred terms – Hyperkalaemia, Blood potassium increased, Hyponatraemia, Blood sodium decreased, Hypotension, Blood pressure decreased, Blood pressure systolic decreased will be summarized separately as other AEs of special interest on-study by severity, serious AESI, study drug related AESI, AESI leading to interruption of study treatment, AESI leading to discontinuation of study treatment, AESI leading to study discontinuation and AESI leading to death.

Data listings will be provided for the following:

- All AEs, all TEAEs and all TE AESIs with dose level at the time of the event, severity, action taken for that AE and outcome of that AE.

4.10.4. Clinical Laboratory Evaluation

Central laboratories will be used for laboratory safety evaluations in this study. Hematology, chemistry, coagulation, urinalysis, UACR, and eGFR will be evaluated. For details regarding the parameters to be included, see Table 4. Laboratory normal ranges will be provided by the central laboratory. An indicator of whether the value is below, within, or above the normal range will represent severity. Laboratory abnormalities will be reported as an abnormal result if the post-baseline result meets abnormality criteria.

Laboratory summaries will be presented for all data after first dose (no cutoff). Quantitative lab results will be summarized in SI units except for UACR and as otherwise indicated in summary outputs. The UACR results will be summarized in mg/g.

In general, laboratory data that are continuous but are less than the LLOQ or above the ULOQ will be imputed as such, and the imputed value will be used for the purpose of calculating summary statistics as specified below:

- A value that is less than the LLOQ or reported in the form of "< x" (where x is considered the LLOQ), x will be multiplied by 0.99 before calculating descriptive statistics. (For example, if the values are reported as < 50 and < 5.0, values of 50 and 5.0 will be

multiplied by 0.99, hence 49.5 and 4.95, respectively, will be used to calculate summary statistics.

- A value that is above the ULOQ will be multiplied by 1.01 before calculating descriptive statistics if the value is reported in the form of "> x" (where x is considered the ULOQ).
- The above rule will be used before calculating descriptive statistics if LLOQ or ULOQ value is reported in the form of "≤ x" or "≥ x" (where x is considered the LLOQ or ULOQ).

Laboratory data collected during the study will be analyzed and summarized using both quantitative and qualitative methods based on the safety population.

A listing for laboratory test results will be provides by PID, and timepoint in chronological order for chemistry, hematology, coagulation, and urinalysis separately. Values falling outside of the relevant reference range will be flagged in the data listings, as appropriate.

To summarize numeric laboratory results, descriptive statistics will be provided by treatment group for each laboratory test specified in the CSP Appendix B as follows:

- Baseline values.
- Values at each post baseline visit.
- Change from baseline at each post-baseline visit.

A baseline laboratory value will be defined following the method described in Section 4.3. Change from baseline to a post-baseline visit will be defined as the visit value minus the baseline value.

The laboratory abnormality will be marked TE if the abnormality occurs during the double-blind treatment period from Day 1 onwards. The summary (number and percentage of patients) for TE laboratory abnormalities will be provided by central laboratory test and treatment group by visit. The denominator is the number of patients with non-missing post-baseline values. All available laboratory values from both scheduled and unscheduled visits after Day 1 (including values both on or off treatment) will be included in the evaluation of abnormalities.

The proportion of patients with > 30% reduction in eGFR and proportion of patients with > 50% increase in serum creatinine at each scheduled assessment visit from previous visit, and proportion of patients achieved overall > 30% and > 50% reduction in eGFR on-study (including unscheduled visits) will be summarized by treatment groups.

The proportion of patients with overall potassium value at any post-baseline (scheduled or unscheduled)visit value ≥ 5.5 mEq/L or ≥ 6.0 mEq/L will be summarized separately for the

following subgroups: eGFR (< 45, ≥ 45), eGFR (30, 30 - <45, 45 - < 60 , ≥ 60), Type 2 diabetes at baseline (yes or No), diuretic use at baseline (yes or No), SGLT2i use, baseline potassium (<= 4.5 vs > 4.5), baseline potassium (<= 4.0, >4.0 - 4.5, > 4.5 - 4.8, > 4.8) and Glucagon-Like Peptide-1 Receptor Agonists used at baseline (Yes or No).

A by-patient listing of on-study laboratory abnormalities will also be provided by treatment group, PID and visit. This listing will include all test results that were collected throughout the study for the laboratory test of interest, with all applicable abnormal flags displayed.

A shift table comparing baseline to maximum or minimum post-baseline values in terms of normal range (low, normal, high, or missing) will be produced, presenting numbers and percentages in each intersection of baseline/post-baseline categories.

The key patient information for potential Hy's Law patients will be summarized.

Change in serum potassium and sodium levels from baseline to Week 26 between each dose strength of CIN-107 compared to placebo will be analyzed using an MMRM model at all planned post-baseline scheduled assessments up to Week 26 (Visit 11). The analysis will include fixed effects for treatments (CIN-107 treatment strategies and placebo), visit, stratification variables (SGLT2 inhibitor use, baseline SBP category and CKD category), the treatment-by-visit interaction, along with a covariate of the baseline potassium/sodium value and baseline potassium/sodium by visit interaction.

The least square means, standard errors for each treatment (CIN-107 and placebo) and the estimated treatment differences for the treatment comparisons (CIN-107 vs placebo group) will be summarized together with 95% CIs, along with p-values for the treatment comparisons for all scheduled visits until Week 26. The effect at Week 26 will be tested with 0.05 2-sided alpha. All other p-values will be nominal.

In addition, below plots based on the safety population will be provided:

- Box plots of potassium and sodium concentrations by each treatment group for each visit.
- Separate spaghetti plots of potassium level by analysis day for patients that had potassium measure in the following categories at any post-baseline visit: ≥ 5.1 mEq/L, ≥ 5.5 mEq/L; ≥ 6.0 mEq/L.
- eDISH plots for each patient.
- Mean and associated 95% CI for the change from baseline in serum potassium by each visit.
- Mean and associated 95% CI for the change from baseline in serum sodium by each visit.

Table 4: Laboratory Variables

**Standard Safety Chemistry Panel**	
Alanine aminotransferase<br>Alkaline phosphatase<br>Aspartate aminotransferase<br>Blood urea nitrogen<br>Chloride<br>Creatinine<br>Gamma-glutamyl transferase<br>Inorganic phosphorus<br>Lipase<br>Sodium<br>Total protein	Albumin<br>Amylase<br>Bicarbonate<br>Calcium<br>Creatine kinase<br>Estimated glomerular filtration rate<br>Glucose<br>Lactate dehydrogenase<br>Potassium<br>Total bilirubin<br>Uric acid
**Additional Chemistry Parameters**	
Glycosylated hemoglobin (HbAl)	
**Hematology**	
Hematocrit<br>Platelets<br>White blood cell count and differential [a]	Hemoglobin<br>Red blood cell count
**Coagulation**	
Activated partial thromboplastin time<br>International normalized ratio	Prothrombin time
**Urinalysis**	
Bilirubin<br>Glucose<br>Leukocyte esterase<br>Nitrite<br>Protein<br>Urobilinogen	Blood<br>Ketones<br>Microscopy [b]<br>pH<br>Specific gravity
**Additional Urinalysis Analytes**	
Sodium<br>Potassium<br>Creatinine	Chloride<br>Phosphate<br>Albumin
**Endocrinology**	

Follicle-stimulating hormone (FSH)	β -human chorionic gonadotropin
**Serology**	
Hepatitis B surface antigen<br>Human immunodeficiency virus antibody	Hepatitis C virus RNA

a Manual microscopic review is performed only if white blood cell count and/or differential values are out of reference range.

b Microscopy is performed only as needed based on positive dipstick test results.

FSH levels will be measured only for female patients who are postmenopausal for at least 1 year at screening and are not surgically sterile.

Serum or point-of-care pregnancy tests will be performed only for female patients of childbearing potential (ovulating, premenopausal, and not surgically sterile).

4.10.5. Physical Examination findings

A complete physical examination will be performed at screening and limited examination is done Physical examination findings will be listed only.

4.10.6. Vital Signs

Changes from baseline in each vital signs (standing and seated SBP and DBP, pulse, respiratory rate, temperature, weight, and BMI) to each corresponding post-baseline assessment will be calculated. Absolute values and changes from baseline will be summarized for all patients by actual treatment group and by visit.

There will be no imputation for missing values. Observed values and changes from baseline will be compared to the relevant AZ defined reference ranges for vital signs (see Table 5) and clinically important change criteria and all values (observed and change) falling outside the reference ranges will be flagged in the listings.

The proportion of patients with low BP at any time on study will be summarized by SBP < 100 mmHg and SBP < 90 mmHg.

Table 5: Vital Signs Predefined Criteria

Vital sign (unit)	Level	Observed value	Notable change from baseline
SBP (mmHg)	High	≥ 140	Increase of ≥ 20
	Low	< 90	Decrease of ≥ 20
DBP (mmHg)	High	≥ 90	Increase of ≥ 10
	Low	< 60	Decrease of ≥ 10

4.10.7. ECG Results

Descriptive statistics (mean, SD, median, Q1, Q3, minimum and maximum) will be provided by actual treatment group and by each visit for 12-leads ECGs including HR, PR interval, RR interval, QRS interval, QT interval and QTcF duration.

A shift table of ECG results at each visit compared with baseline values will be presented by treatment group using the following categories: normal; abnormal (not clinically significant); abnormal (clinically significant); or missing. The number and percentage of patients in each cross-classification group of the shift table will be presented. Patients with a missing value at baseline will not be included in the denominator for percentage calculation. No formal statistical testing is planned.

Treatment emergent QTcF abnormalities On-study will be summarized for the following categories for each treatment group:

QTcF value at any time during study: > 450 (msec), > 480 (msec) and > 500 (,sec).

QTcF increase from baseline at any time during study: > 30 (msec), > 60 (msec)

QTcF value and QTcF increase from at any time during study:

- QTcF Value > 450 (ms) and increase >30(ms) [b]
- Value >500 (ms) and increase >60 (ms) [b]

The listing for ECG assessment results will be provided by treatment group, Patient ID and by visit. Clinically significant abnormal results will be flagged.

4.10.8. Pharmacokinetics

Plasma concentrations-time data will not be analysed to determine the PK parameters. The PK concentration data will be reviewed combined with dosing, concomitant medication and AE data on a case-by-case bases to determine whether any exclusion from the PK analysis set is deemed necessary.

Plasma concentration-time data will be presented using PK analysis set (see Section 4.5.3) and will be summarized by actual starting dose of CIN-107 received. PK concentration data will be listed for each patient, together with the flag and reason for those excluded from PK analysis. Plasma concentrations will be summarized by treatment by analysis visit, by means of descriptive statistics as follows:

- Geometric mean (gmean, calculated as exp[μ], where μ is the mean of the data on a logarithmic scale).
- Coefficient of variation (gCV%, calculated as 100 ($\sqrt{\exp(s^2) - 1}$) where s is the standard deviation of the data on a log scale).
- Gmean ± geometric standard deviation (gmean+gSD and gmean-gSD), calculated as exp[μ ± s]).
- Mean.
- SD.
- Median.
- Minimum (min).
- Maximum (max).
- Number of observations (n).
- n > LLOQ.

Plasma concentrations that are BLQ will be reported as follows:

- Individual concentrations below the LLOQ of the bioanalytical assay will be reported as NQ in the listings with the LLOQ defined in the footnotes of the relevant TFLs.
- Individual plasma concentrations that are Not Reportable will be reported as NR and those that are missing will be reported as NS (No Sample) in the listings. Plasma concentrations that are NQ, NR, or NS will be handled as follows for the provision of descriptive statistics:
- Any values reported as NR or NS will be excluded from the summary tables and figures. At a time point where less than or equal to 50% of the concentration values are NQ, all NQ values will be substituted with the LLOQ concentration, and all descriptive statistics will be calculated accordingly.
- At a time point where more than half (but not all) of the values are NQ, mean, gmean, gCV%, +gSD and SD will be set to NC. The maximum value will be reported from the individual data and the minimum and median will be set to NQ.
- If all concentrations are NQ at a time point, the mean, gmean, min, median and max will be reported as NQ and the +gSD, gCV%, and SD will be reported as NC.

The number of values above LLOQ (n > LLOQ) will be reported for each time point together with the total number of collected values.

Three observations > LLOQ will be required as a minimum for PK plasma concentrations to be summarized. Two values will be presented as minimum and maximum with the other summary statistics as NC.

4.10.9. Pharmacodynamics

Central laboratories will be used for serum/plasma PD parameters and 24hr urine PD parameters evaluations in this study. For details regarding the parameters to be included, see Table 6 and Table 7.

In general, pharmacodynamic data that are continuous but are less than the LLOQ or above the ULOQ will be imputed as such, and the imputed value will be used for the purpose of calculating summary statistics as specified below:

- A value that is less than the LLOQ or reported in the form of "< x" (where x is considered the LLOQ), x will be multiplied by 0.99 before calculating descriptive statistics. (For example, if the values are reported as < 50 and < 5.0, values of 50 and 5.0 will be multiplied by 0.99, hence 49.5 and 4.95, respectively, will be used to calculate summary statistics.
- A value that is above the ULOQ will be multiplied by 1.01 before calculating descriptive statistics if the value is reported in the form of "> x" (where x is considered the ULOQ).
- The above rule will be used before calculating descriptive statistics if LLOQ or ULOQ value is reported in the form of "≤ x" or "≥ x" (where x is considered the LLOQ or ULOQ).

Table 6 Pharmacodynamic Analytes — Blood Sample

Serum or plasma aldosterone and its precursors (18-OH- corticosterone, corticosterone, and 11-deoxycorticosterone)	Total cortisol and its precursor 11-deoxycortisol
Plasma renin activity (PRA)<br>N-terminal pro-B-type natriuretic peptide (NT-proBNP)<br>Angiotensinogen<br>Serum PICP	Aldosterone/PRA ratio<br>Direct renin concentration<br>Angiotensin II<br>The ratio of procollagen type-I C-terminal propeptide to collagen type-1 C-terminal telopeptide

C = carboxy; NT-proBNP = N-terminal pro-B-type natriuretic peptide; PICP = procollagen type-I C-terminal propeptide; PRA = plasma renin activity

Table 7 Pharmacodynamic Analytes — 24-Hour Urine Collection

Albumin	Neutrophil gelatinase-associated lipocalin (NGAL)

Aldosterone	Potassium
Creatinine	Protein
Cystatin C	Renin
Sodium	Kidney injury molecule-1

PD data collected during the study will be analyzed and summarized by quantitative methods based on the Pharmacodynamic population.

A listing for PD test results will be provides by patient ID, and timepoint in chronological order for serum/plasma and 24hr urine separately. Values falling outside of the relevant reference range will be flagged in the data listings, as appropriate.

To summarize numeric results, descriptive statistics will be provided by treatment group for each PD parameter test specified as follows:

- Baseline values.
- Values at each post baseline visit.
- Change from baseline at each post-baseline visit.

A baseline laboratory value will be defined following the method described in Section 4.3. Change from baseline to a post-baseline visit will be defined as the visit value minus the baseline value (see Section 4.8.5).

4.10.10. Pregnancy Test

Both serum and urine pregnancy test will be done for female patients. The pregnancy test results will be listed for all female patients fulfilling the childbearing potential, by visit. If pregnancy test is not performed for a female patient, the reason for not doing the test will be listed.

4.11. Interim Analysis

No formal interim analysis is planned.

4.12. Data Safety Monitoring Board

An independent DSMB will be formed to conduct data reviews to assess safety and tolerability of CIN-107 and to make recommendations to AZ regarding the study. Details regarding DSMB responsibilities, authorities, and procedures, etc. are documented in the DSMB Charter. The

tables, listings, and figures to be provided to the DSMB closed session are documented separately and will follow the methods described in Section 4.

4.13. Unblinded Review Committee

The Sponsor may initiate an URC, whose members are not involved in the execution of the study and would be described in a separate URC Charter. The URC would assess the safety and efficacy of baxdrostat in a CKD population in preparation for the initiation of Phase III studies. Review by the URC is not intended to inform any decision making by AZ regarding the conduct of this ongoing Phase II trial D6972C00001. The Sponsor will take every necessary step to maintain the scientific and data integrity of this study.

4.14. Changes to Protocol-planned Analyses

Not applicable to this study.

4.15. Sample Size Determination

The sample size for the study was planned to adequately power the study for the primary endpoint and first 2 secondary efficacy endpoints. Assuming an early withdrawal rate of 8% and a common standard deviation of 11 mmHg, with 300 randomized patients (randomization ratio of 1:1:1, 100 patients in the low dosing strategy, high dosing strategy, or placebo group), the study would have 95.7% power to detect a 6 mmHg difference in change from baseline in SBP between a CIN-107 dosing strategy group versus the placebo group at Week 26 and 98.9% power to detect a difference between the pooled CIN-107 group and a placebo group using a 2-sided significance level of 0.05.

Based on AZ recalculation of sample size, the protocol was amended to stop further enrollment of patients. The decision was made in the absence of Sponsor review of unblinded interim results. Randomization of eligible patients from the pool of patients in screening or run-in after stopping new enrollments will be permitted, resulting in approximately 174 patients randomized (randomization ratio of 1:1:1 with approximately 53 patients each in the low dosing strategy, high dosing strategy, or placebo group), sufficient for 90% power for the primary endpoint assuming a treatment difference of 6 mmHg and a standard deviation of 11 mmHg and a dropout rate of 8%.

5. SUPPORTING DOCUMENTATION

Appendix 1: List of Abbreviations

Abbreviation/Acronym	Definition/Expansion
ACEi	Angiotensin-converting enzyme inhibitor
AE	Adverse event
AESI	Adverse event of special interest
ANCOVA	Analysis of covariance
AOBPM	Automated office blood pressure monitoring
ATC	Anatomical Therapeutic Chemical
AR	Autoregressive model
ARB	Angiotensin receptor blocker
ARR	Aldosterone renin ratio
AZ	AstraZeneca
BMI	Body mass index
BP	Blood pressure
bpm	Beats per minute
BLQ	below the limit of quantification
CI	Confidence interval
CKD	Chronic kidney disease
CS	Compound symmetry
CSP	Clinical study protocol
CTX-1	collagen type 1 C-terminal telopeptide
CV	Coefficient of variation
DBP	Diastolic blood pressure
DSMB	Data Safety Monitoring Board
ECG	Electrocardiogram
eCRF	Electronic case report form
eDISH	Evaluation of Drug-Induced Serious Hepatotoxicity
eGFR	Estimated glomerular filtration rate
EOT	End of treatment
FSH	Follicle-stimulating hormone
FU	Follow-up
gmean	Geometric mean
GMR	Geometric Mean Ratio
gSD	Geometric standard deviation
gCV	Geometric coefficient of variation

Abbreviation/Acronym	Definition/Expansion
HbA1	hemoglobin
HR	Heart rate
ICH	International Council for Harmonisation
IPD	Important protocol deviation
ICE	Intercurrent event
IPD	Important protocol deviation
IRT	Interactive response technology
ITT	Intent-to-Treat
KIM-1	kidney injury molecule-1
LLOQ	Lower limit of quantitation
MedDRA	Medical Dictionary for Regulatory Activities
mITT	Modified Intent-to-Treat
MMRM	Mixed-effect model for repeated measures
MNAR	Missing not at random
NGAL	neutrophil gelatinase-associated lipocalin
NQ	Not quantifiable
NR	Not reported
NS	No sample
NT-proBNP	N-terminal pro-B-type natriuretic peptide
PD	Pharmacodynamic
PICP	procollagen type-I carboxy-terminal propeptide
PID	Patient/participant identification
PK	Pharmacokinetic
PRA	Plasma renin activity
PXL	PAREXEL
Q1	First quartile
Q3	Third quartile
QTcF	QT interval corrected using Fridericia's formula
REML	Restricted maximum likelihood
RNA	Ribonucleic acid
SAE	Serious adverse event
SAP	Statistical analysis plan
SBP	Systolic blood pressure
SD	Standard deviation
SGLT2	Sodium-Glucose Transport Protein 2
SOC	System Organ Class
SOP	Standard operating procedures

Abbreviation/Acronym	Definition/Expansion
TE	Treatment-emergent
TEAE	Treatment-emergent adverse event
TESAE	Treatment-emergent serious adverse event
UACR	Urinary albumin-to-creatinine ratio
uHTN	Uncontrolled Hypertension
ULOQ	Upper limit of quantification
WHO	World Health Organization

6. REFERENCES

Signature Page for VV-RIM-04860957 v3.0

Approve: Document Level Task<br>Verdict: Approved	[redacted]

Signature Page for VV-RIM-04860957 v3.0
